# Historical Drugs in Transylvania: Disclosing the Composition of Ointments from the “History of Pharmacy Collection” in Cluj-Napoca Through a Multi-Analytical Approach

**DOI:** 10.3390/molecules29225356

**Published:** 2024-11-14

**Authors:** Federica Nardella, Jacopo La Nasa, Ilaria Degano, Francesca Modugno, Ana-Maria Gruia, Ioana Cova, Andrea Beatrix Magó, Márta Guttmann, Erika Ribechini

**Affiliations:** 1 Department of Chemistry and Industrial Chemistry, University of Pisa, I-56123 Pisa, Italy; jacopo.lanasa@unipi.it (J.L.N.); ilaria.degano@unipi.it (I.D.); francesca.modugno@unipi.it (F.M.); erika.ribechini@unipi.it (E.R.); 2 National Museum of Transylvanian History, 400020 Cluj-Napoca, Romania; ana.gruia@gmail.com (A.-M.G.); ioanatescancova@yahoo.com (I.C.); beatrix.b3@gmail.com (A.B.M.); 3 Department of History, Heritage and Protestant Theology, Faculty of Social and Human Sciences, “Lucian Blaga” University of Sibiu, 550024 Sibiu, Romania; marta.guttmann@ulbsibiu.ro

**Keywords:** pharmaceutical historical ointments, gas chromatography–mass spectrometry, analytical pyrolysis, solid-phase microextraction, liquid chromatography/mass spectrometry, PHARMATRANS project

## Abstract

The National Museum of Transylvanian History in Cluj-Napoca, Romania, features a History of Pharmacy Collection that documents the evolution of pharmacies in the region since the 16th century. Within the “Pharmatrans” project (2021–2023), we investigated the chemical composition of ointments from fourteen historical pharmaceutical containers dating back to the 18th and 19th centuries. Most samples were from an aristocratic traveling medicine chest, a key artifact in the collection. This study marks the first extensive analysis of historical pharmaceutical formulations in Romania, enhancing our understanding of these valuable items. The main ingredients of formulations were characterized using gas chromatography–mass spectrometry (GC–MS), solid-phase microextraction–GC–MS (SPME–GC–MS), and pyrolysis–GC–MS (Py–GC–MS). Additionally, high-performance liquid chromatography coupled with high-resolution mass spectrometry (HPLC-ESI-Q-ToF) was employed for the detailed analysis of lipid materials and polar compounds. Elemental analysis was conducted using field emission gun–scanning electron microscope (FEG–SEM) with energy-dispersive spectroscopy (EDS). The results revealed that twelve out of fourteen mixtures contained interpretable organic content, often aligning with the vessels’ labels. The findings indicate that Transylvanian elites in the late 18th century had access to both rare drugs and traditional remedies, reflecting contemporary trends in pharmacy.

## 1. Introduction

Pharmacists in Cluj-Napoca had expressed an interest in establishing a museum dedicated to the history of pharmacies and pharmaceuticals in the city since mid-19th century. Still, the first History of Pharmacy exhibition opened to the public in only 1917, starting from a core collection of artifacts gathered from “historical Hungary” (present-day Transylvania, Hungary, Slovakia). Two additional larger lots were subsequently incorporated, some of which were previously displayed for educational purposes at the local pharmaceutical institute, while some others consisted of items collected from Transylvanian pharmacies nationalized in 1949 by the Communist State [1]. In 1954, the reunited collections were exhibited in the best-known pharmacy in Cluj, named “Dr. Hintz”. The collection is the oldest in Romania and one of the most valuable, rivaling that of Sibiu and surpassing those in Sighișoara and Oradea.

One of the most treasured artifacts of the core collection is an 18th-century medicine chest that once belonged to countess Terezia Kemény (as attested by a manuscript invoice preserved inside). The chest preserves almost all its parts (inner compartments, sliding covers and doors, containers, paper envelopes) and, most importantly, a significant variety of *materia medica* [2].

The Hintz house is a historical building with a pharmacy active between about 1750 and 1949, which has hosted the History of Pharmacy collection since 1954. In 2018–2023, the pharmacy went through a major restoration that involved the temporary relocation of the entire collection. Considering the importance and value of the collection, during the renovation of the building a thorough study, conservation and cataloguing of the objects pertaining to the History of Pharmacy Collection was planned, taking into consideration more than 3500 individual items recorded under about 2500 inventory numbers. Until this recent endeavor, the only existing catalogue of the collection, published in 1996, was partial, only including a few poor-quality black-and-white reproductions, and was available in French only. Further, very limited and scattered data can be found in few articles and studies [3,4,5,6]. None of the materials have been chemically characterized before, while the old books and more than half of the manuscripts have never been published (edited or translated). The 2018–2023 renovation also led to the discovery of artifacts that lacked inventory numbers, and historical finds, such as a trove of 19th–20th-century pharmaceutical goods excavated in the courtyard. A total of 1069 artifacts were also inventoried in 2020–2023, representing almost 30% of all goods in the collection.

In this framework, the project “PHARMATRANS—All things Apothecary in 16th–20th-century Transylvania. The History of Pharmacy Collection in Cluj-Napoca” funded for the period 2021–2023 by the Romanian Ministry of Education and Research (UEFISCD) entailed the publication in English of a complete catalogue of the collection, published in November 2023 and available online (https://pharmatrans.mnit.ro/en/catalogue/ (accessed on 6 November 2024)) [1]. The first volume contains various introductory studies, while the next five volumes present all the items of the collection, divided among containers made of different materials (II. Wooden Containers; III. Pottery and Metal Containers; IV. Glass Containers), or written sources (V. Manuscripts; VI. Books), while the seventh volume is dedicated to the Varia Pharmaceutica (other apothecary artifacts and few non- apothecary related artifacts).

The project also entailed the conservation and restoration of around 500 miscellaneous objects, the research of the history of pharmacy in Transylvania during the 18th century based on the previously unpublished sources in the collection (manuscripts, jars, tools, printed materials), the transcription of some of the manuscripts in the collection, and the chemical characterization of the ointments contained in the most valuable items of the collection.

The chemical investigation of historical ointments holds significant importance as it provides insights into the medicinal, pharmacological and cosmetical, and thus technological, evolution of society through history, in different social and geographical environments [7]. The chemical characterization of ingredients and formulations of ancient pharmaceutical and cosmetic preparations is fundamental for the understanding of the ointments’ original preparations and uses, and their significance in the historical context [8,9]. However, the chemical analysis of these archaeological and historical artifacts is challenging as they were composed of mixtures of natural substances extracted from different sources [10,11]. The final formulations result in quite complex mixtures, not only due to the multiple ingredients employed, but also because of the chemical modifications induced during preparation, or by ageing, influenced also by the possible interactions among the materials. For these reasons, the investigations require a wide array of analytical methodologies that allow us to disclose each material employed. The main analytical strategies to characterize historical pharmaceutical ointments are based on spectroscopic and chromatographic techniques. In particular, gas chromatography, high-performance liquid chromatography coupled with mass spectrometry and analytical pyrolysis–gas chromatography–mass spectrometry (GC–MS, HPLC/MS and Py–GC–MS) have been employed in the study of ointments to reveal several classes of organic compounds such as acyl-lipids, waxes and resins [9,11,12,13]. Recently, headspace analysis has also been performed on ointments by solid-phase micro-extraction (SPME) followed by GC–MS to determine the presence of volatile organic compounds [14].

This research aimed at identifying the composition of the ointments collected from fourteen historical containers belonging to the collection of the National Museum of Transylvanian History (Cluj-Napoca) and at reconstructing the pharmaceuticals available in the 18th century to the Transylvanian upper classes, in terms of materials and recipes. In order to achieve such goal, a multi-analytical approach was adopted, comprising gas chromatography–mass spectrometry (GC–MS) and analytical pyrolysis–gas chromatography–mass spectrometry (Py–GC–MS) to identify the main organic ingredients, solid-phase microextraction–gas chromatography–mass spectrometry (SPME–GC–MS) to profile the volatile fraction of the samples, high-performance liquid chromatography coupled with high resolution mass spectrometry (HPLC-ESI-Q-ToF) to characterize acyl-lipids and waxes along with small polar compounds, and FEG–SEM equipped with an energy-dispersive spectrometer (EDS) for X-ray microanalysis to perform elemental chemical analysis and obtain information on inorganic components.

## 2. Results and Discussion

To obtain a comprehensive overview of the ingredients used in the formulations of the ointments, we applied a multi-analytical approach that allowed us to characterize their molecular composition. In particular, all the samples were first analyzed by GC–MS and, and then, on the basis of this preliminary information and if the sample was still available, other analytical techniques were applied to obtain more specific information.

Table 1 summarizes the results obtained for each formulation, while the following paragraphs give a detailed description of the analytical results. Lipids were highlighted as the most common material class in the ointment formulations, followed by resinous and polysaccharide substances. In addition, the study of the composition also demonstrated the presence of tannins in several ointments. Finally, the inorganic constituents were also studied, showing that several relevant elements, such as gold and lead, were contained in the ointment formulations. The following discussion is based on the main material chemically identified and on the labels on the jars.

### 2.1. Lipid and Polysaccharide Materials

The methods based on gas and liquid chromatography coupled with mass spectrometry allowed us to identify markers of oils and fats in samples IF 1908, IF 1914, IF 1918 and IF 1906.

**Figure 1:**
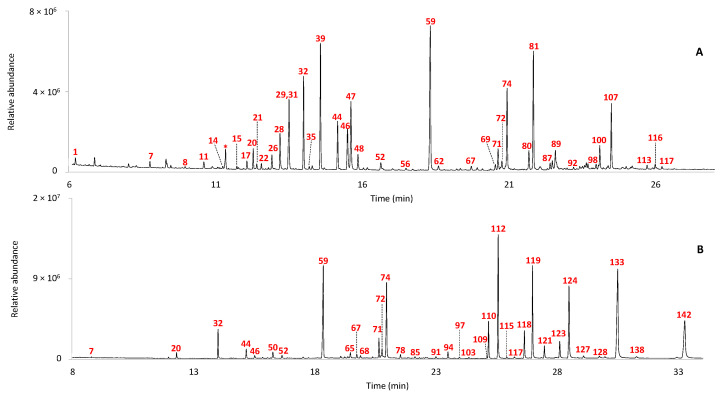
GC–MS chromatograms of the (**A**) acidic fraction and (**B**) neutral fraction obtained after alkaline hydrolysis and solvent extraction of sample IF 1908. The peaks are labeled according to Table 2. (*): contaminants present in the analytical blanks.

For example, Figure 1 shows the GC–MS chromatograms of the extracts obtained after alkaline hydrolysis, solvent extraction and derivatization of sample IF 1908. The GC–MS chromatograms obtained for all the ointments are included in the Supplementary Material. The chromatographic peaks are labelled according to Table 2.

The chromatographic profile of IF 1908 can be considered representative of samples featuring the molecular markers of lipid materials such as vegetable oils, animal fat or beeswax that were extensively used as carriers, excipients or stabilizers for the preparation of pharmaceuticals.

The GC–MS chromatographic profile of the acidic fraction of sample IF 1908, supposedly made of turpentine, myrrh and galbanum, shows peaks ascribed to saturated fatty acids ranging from 10 to 24 carbon atoms, with palmitic acid (#59) as the most abundant. The GC–MS analysis allowed us also to detect numerous oxidation products of polyunsaturated glycerolipids such as α,ω-dicarboxylic acids (ranging from 2 to 11 carbon atoms), hydroxyacids and dihydroxyacids. Azelaic acid (α,ω-nonanoic acid, #39) is the most abundant among the α,ω-dicarboxylic acids. This compound is known to be an oxidation product of vegetable oils characterized by polyunsaturated acyl chains [11]. In particular, the predominance of azelaic acid amongst the α,ω-dicarboxylic acids implies the presence of an unsaturation in position 9 of the original unsaturated fatty acid, as it occurs in oleic, linoleic and linolenic acids. This result is consistent with the occurrence of the chromatographic peaks of the diastereomers of 9,10-dihydroxyoctadecanoic acid (#98 and #100), which can be produced via the hydroxylation of the unsaturated acids in correspondence of the double bond in position 9. Table 3 shows the profile in terms of relative percentages of the constituting fatty acids of the four samples featuring a significant amount of fatty acids after saponification.

Sample IF 1908 contains a relatively higher level of dicarboxylic acids (α,ω-Cx %, Table 3) compared to the other samples that were rich in glycerolipids, and this could be due to a higher oxidation level of this sample, or to the presence of polyunsaturated lipids in the ointment original formulation.

The GC–MS chromatogram in Figure 1A also shows the presence of 14- and 15-hydroxyhexadecanoic acids (#80 and #81), which are known to be molecular markers of beeswax [15,16]. The presence of beeswax is confirmed by the peaks corresponding to long chain alcohols, diols and alkanes in the chromatogram of the neutral fraction reported in Figure 1B (#112, #119, #121, #124, #127, #133 and #142) [17]. The presence of beeswax may explain the high value of the relative percentage content of palmitic acid in the samples.

In addition, the GC–MS profile of sample IF 1908 features the presence of diterpenoid acids: didehydroabietic (#87), dehydroabietic (#89), 7-oxodehydroabietic (#107) and 15-hydroxy-7-oxodehydroabietic acids (#116). These compounds are makers for a plant resin obtained from Pinaceae trees [18], consistently with the hypothesized recipe of the balm contained in the jar, based on turpentine.

**Figure 2:**
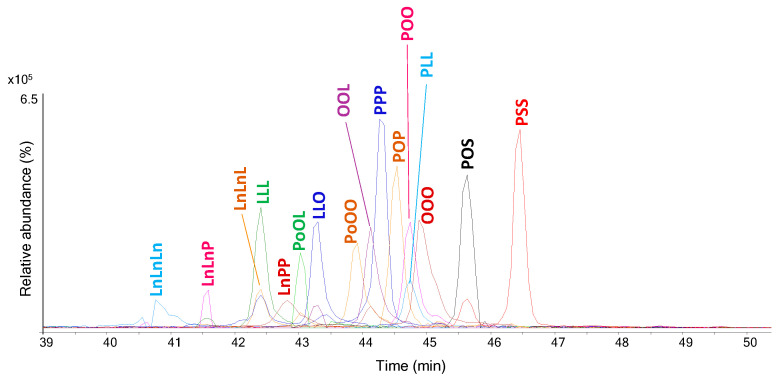
EIC profiles obtained from HPLC–MS/MS analysis of the extract of sample IF 1908. Profiles are obtained by overlapping EICs corresponding to the *m/z* of [M+Na]^+^ reported in the literature [19] for the different species. Labels correspond to the fatty acids constituting the following triacylglycerols: La, lauric acid (C12:0); M, myristic acid (C14:0); P, palmitic acid (C16:0); Po, palmitoleic acid (C16:1); S, stearic acid (C18:0), O, oleic acid (C18:1); L, linoleic acid (C18:2); Ln, linolenic acid (C18:3).

To further confirm the presence of oil/fats and to provide unambiguous information on the origin of the lipids, the sample was analyzed by HPLC–MS/MS for the direct identification of triacylglycerols (TAGs) [11]. Figure 2 shows the extracted ion chromatograms (EICs) corresponding to the *m/z* of [M+Na]^+^ ions for the most abundant triacylglycerols detected for sample IF 1908 [11].

The EIC profiles show the presence of TAGs with saturated acyl chains (palmityl and stearyl) that can be considered indicative of the presence of animal fats from ruminants [18], together with the presence of TAGs containing polyunsaturated acyl chains (linoleyl and linolenyl) that are characteristic of a vegetable oil with siccative properties [19]. Moreover, the HPLC–MS/MS analysis allowed us to confirm the presence of beeswax by the detection of high molecular weight species such as mono-, di-, tri- and tetra-esters (Figure S1 of the Supplementary Material).

Sample IF 1908 was also analyzed by SPME–GC–MS to evaluate the presence of volatile organic compounds that could not be detected by the previous analytical approaches. The SPME–GC–MS chromatogram showed the presence of a high abundance of camphor and verbenone. These two terpenes are known to be used in medicinal preparations and can occur in many aromatic herbs such as rosemary, sage, licorice and mint [20]. Moreover, the headspace of the sample was characterized by the presence of many short chain fatty acids, aldehydes and alkanes, consistent with the already hypothesized presence of beeswax and lipid materials and the oxidation processes of fatty acids containing polyunsaturated acyl chains [21].

The GC–MS chromatogram of the acidic fraction of sample IF 1918 (Supplementary Material, Figure S2) is dominated by the peak of oleic acid (#71), which suggests the presence of a relatively well-preserved and non-oxidized vegetable oil. In fact, the relative percentage of oleic acid (C18:1%, Table 3) is much higher than what is expected in aged oils/fats from archaeological samples, being the double bond of oleic acid relatively reactive towards oxidation [18]. For this reason, it is possible to hypothesize that the material in this vessel was protected from exposure to light and oxygen. The label on this vessel being absent, no correlation between a hypothetical recipe with the chemical evidence can be suggested for this ointment. To further characterize the lipid substance, sample IF 1918 was analyzed by HPLC–MS/MS; however, the TAG signals obtained in the HPLC–MS/MS chromatogram were not present in significant amounts (traces of TAGs were at procedure blank level). The absence of significant amounts of specific TAGs did not allow us to unambiguously characterize the type of lipid material in IF 1918. For further investigation, this sample was also subjected to Py–GC–MS in order to evaluate the possible presence of polymeric substances not evidenced by GC–MS and HPLC/MS as lignocellulosic polymers or a polysaccharidic material, such as gums or honey [18]. The pyrogram reported in Figure S3 of the Supplementary Material showed the presence of several sugars and anhydrosugars such as levoglucosan (1,6-anhydro-β-D-glucopyranose), 1,4-anhydro-β-D-galactopyranose, 1,6-anhydro-β-D-glucofuranose, 1,5-anhydroglucitol, 3-deoxy-D-ribo-hexonic acid, γ-lactone and 3-deoxy-D-arabino-hexonic acid, γ-lactone along with secondary pyrolysis products of poly/oligosaccharides. This result demonstrated that saccharide/polysaccharide materials, such as gums or honey, were ingredients of the ointment [22].

The fatty acid profile of sample IF 1914 (GC–MS chromatograms in the Supplementary Material, Figure S4) also showed a series of compounds ascribable to glycerolipids, but in this case the presence of cholesterol (#125), not observed in the two samples discussed above, suggests that this sample contains an animal fat. Due to the limited amount of sample available, further analysis could not be carried out to confirm the presence of fat or to investigate the occurrence of camphor, mentioned in the label of the jar.

Interestingly, both IF 1914 and IF 1918 show an intense peak of pentachlorophenol (#47), which was used as a pesticide and a disinfectant in the 1930s and can thus be considered an historical environmental contaminant.

**Figure 3:**
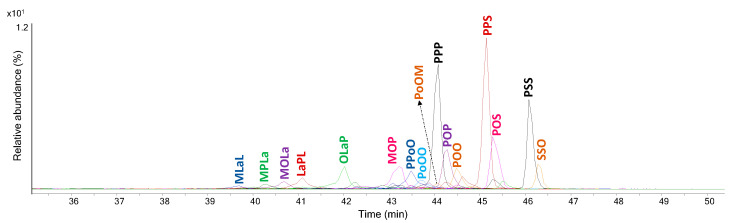
EIC profiles obtained from HPLC–MS/MS analysis of the extract of sample IF 1906. Profiles are obtained by overlapping EICs corresponding to the *m/z* of [M+Na]^+^ reported in the literature [19] for the different species. Labels correspond to the fatty acids constituting the following triacylglycerols: La, lauric acid (C12:0); M, myristic acid (C14:0); P, palmitic acid (C16:0); Po, palmitoleic acid (C16:1); S, stearic acid (C18:0), O, oleic acid (C18:1); L, linoleic acid (C18:2).

The GC–MS chromatographic profile of sample IF 1906 (GC–MS chromatograms in Supplementary Material, Figure S5), which, according to its label, contain olive oil, white wax, spermaceti and lead white, shows the peaks assigned to several saturated and unsaturated fatty acids along with the oxidation products of glycerolipids containing unsaturated acyl substituents. These results confirm the presence of lipid material. In particular, the detection of saturated TAGs in the HPLC–MS/MS profile (Figure 3) suggested its use in the formulation of animal fat. The presence of lipids was also supported by the identification of some volatile degradation products such as short aldehydes and alkanes in the SPME–GC–MS analysis.

Finally, a resin from Pinaceae trees was also detected in the GC–MS profile of sample IF 1906 (Figure S5) on the basis of the detection of the peaks corresponding to the diterpenoid acids didehydroabietic (#87), dehydroabietic (#89), 7-oxodehydroabietic (#107) and 15-hydroxy-7-oxodehydroabietic acids (#116).

FEG–SEM–EDS analysis was also performed on sample IF 1906 to investigate the elemental composition. The image and EDS spectrum obtained for this sample are reported in Figure S6 of the Supplementary Materials. The elemental distribution revealed the presence of lead, a result in accordance with the original recipe of ungv. Album, that included lead white.

### 2.2. Resinous Materials

**Figure 4:**
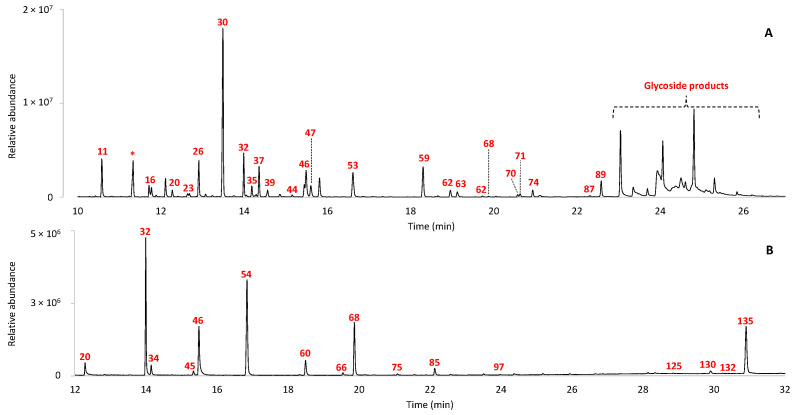
GC–MS chromatograms of the (**A**) acidic fraction and (**B**) neutral fraction obtained after alkaline hydrolysis and solvent extraction of sample IF 1909. The peaks are labeled according to Table 2. (*): contaminants present in the analytical blanks.

Besides IF 1908 and IF 1906 discussed in the previous sections, resinous materials have been identified in several other ointments. The GC–MS chromatograms of sample IF 1909, collected from a jar labelled as “Resin Jallap”, are reported in Figure 4.

The GC–MS chromatographic profiles feature peaks associated with several short chain hydroxyacids (#23, #30, #37) and phenolic acids such as *p*-coumaric acid (#53). In addition, a series of saccharides was detected at high retention times in the chromatogram of the acidic fraction (Figure 4A). The chromatogram of the neutral fraction was instead characterized by the presence of several linear alcohols ranging from 13 to 22 carbon atoms, along with plant sterols, such as campesterol (#130), stigmasterol (#132), β-sitosterol (#135). The occurrence of all these compounds can be related to the use of a ligneous plant material in the formulation, and is consistent with the presence of roots of *Ipomoea purga* (Wender.) Hayne, often used as medicinal plant [23,24,25], and reported as ingredient in the label of the container. Signals related to diterpenoids compounds such as didehydroabietic acid and dehydroabietic acid (#87 and #89) were also identified in the chromatogram of the acidic fraction, suggesting the presence of pine resin. IF 1909 was also analyzed by SPME–GC–MS. The volatile fraction of this sample contained a significant amount of 6-methyl-5-hepten-2-one, also known as sulcatone. Sulcatone has a citrus-like odor, holds antimicrobial activity and is a component of the essential oils of several plants [26]. Based on the literature, there is no evidence of its presence in the chemical composition of jalap root; however, given the structures of the chemical constituents of the root [27], sulcatone could be a degradation product of the original material.

**Figure 5:**
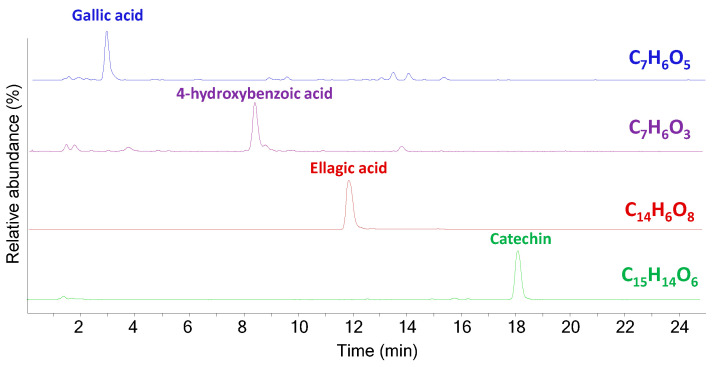
EIC profiles obtained from HPLC–MS/MS analysis of the extract of sample IF 1916 for tannins identification (negative mode).

Ointment IF 1916 (GC–MS chromatograms in the Supplementary Material, Figure S7) was labelled as “Pulvis bezoard”, which according to historical documents corresponds to an agglomeration of undigested matter, encountered mostly in ruminants [28]. The presence of a material of animal origin in this ointment was highlighted by the identification of fatty acids with an odd number of carbon atoms in the acyl chain and by the detection of cholesterol. In addition to these compounds, GC–MS analysis highlighted the presence of peaks corresponding to several pimarane and abietane diterpenoid compounds, such as levopimaric (#76), isopimaric (#77), pimaric (#83), sandaracopimaric (#84), dehydroabietic (#89), abietic acid (#90) and dehydroabietinol (#79). These compounds are all associated with the presence of pine resin. In addition, the peak of ellagic acid (#134) was detected in the GC–MS chromatogram of the acidic fraction, suggesting the possible presence of tannins [29], also confirmed by HPLC–MS/MS analysis. Figure 5 shows the EICs profiles highlighting the presence of gallic acid, 4-hydroxybenzoic acid, ellagic acid and catechin in the extracts of sample IF 1916.

The inorganic components of sample IF 1916 were also investigated by FEG–SEM–EDS analysis. Figures S8–S10 in the Supplementary Materials show the images and EDS spectra obtained for several areas of the amorphous material. The elemental analysis highlighted the presence of calcium, aluminum, iron, zirconium and gold. These results are consistent with some ingredients suggested by the recipe for this ointment that is reported on the label of the container, such as pearl and coral, that are both rich in calcium, ruby (Al_2_O_3_), jacinth (ZrSiO_4_) and gold leaves. In addition, lead, antimony and mercury were also detected in significant amounts. The presence of these elements can be related to the use of ingredients commonly employed in traditional medicine.

The GC–MS chromatograms of sample IF 2405 (GC–MS chromatograms in the Supplementary Material, Figure S11) show the signals of diterpenoid compounds in both the acidic and neutral fraction. In particular, abietane and pimarane acids, along with their oxidation products (#79, #82, #83, #86, #87, #89, #93, #95, #96, #99, #107, #113 and #116) were detected. Their presence is attributed to pine resin. This result was confirmed by SPME–GC–MS analysis. The volatile fraction of IF 2405 is mainly composed of monoterpenoids and sesquiterpenoids typical of resinous materials, that account for about 90% of its organic volatile composition. In particular, the high abundance of longifolene suggests that the pine resin was obtained from Pinus sylvestris, whose resin is particularly rich in this compound [30].

In addition, the GC–MS profile showed the presence of the peak of oleic acid (#71), together with the peaks of saturated fatty acids and oxidation products of glycerolipids, such as α,ω-dicarboxylic acids and dihydroxyacids. These molecular markers suggest the presence in the ointment formulation of a relatively fresh vegetable oil. However, no TAGs above the blank level were detected by HPLC–MS/MS analysis in order to unambiguously characterize the presence of an oil or fat.

The GC–MS chromatograms obtained in the analysis of sample IF 341, labelled as “Pulvis Mumiae” are reported in Figure S12 of the Supplementary Materials. The sample is characterized by the presence of moronic (#145), isomasticadienonic (#151) and masticadienonic acids (#152) that are triterpenoid species known to be molecular markers for mastic resin. The presence of mastic resin was also confirmed by the detection of other triterpenoid compounds such as oleanonic aldehyde (#148), oleanonic acid (#147), oleanane (#136), β-amyrone (#137), ursadienone (#143) and 20,24-epossi-25-idrossidammaren-3-one (#144). In addition, the GC–MS chromatogram of the acidic fraction shows the peak of p-coumaric acid (#53), confirming the presence of a plant-derived material. The presence of resinous materials was also confirmed by the detection of mono- and sesqui-terpenoids in the profile of the volatiles characterized by SPME–GC–MS analysis. These results are consistent with the recipe of the ointment suggested by the label, since all the materials identified in the sample were widely used as embalming ingredients.

Finally, traces of pine resin were identified in sample IF 1857 (Figure S13) by detecting the peak of dehydroabietic acid (#89).

### 2.3. Other Materials

**Figure 6:**
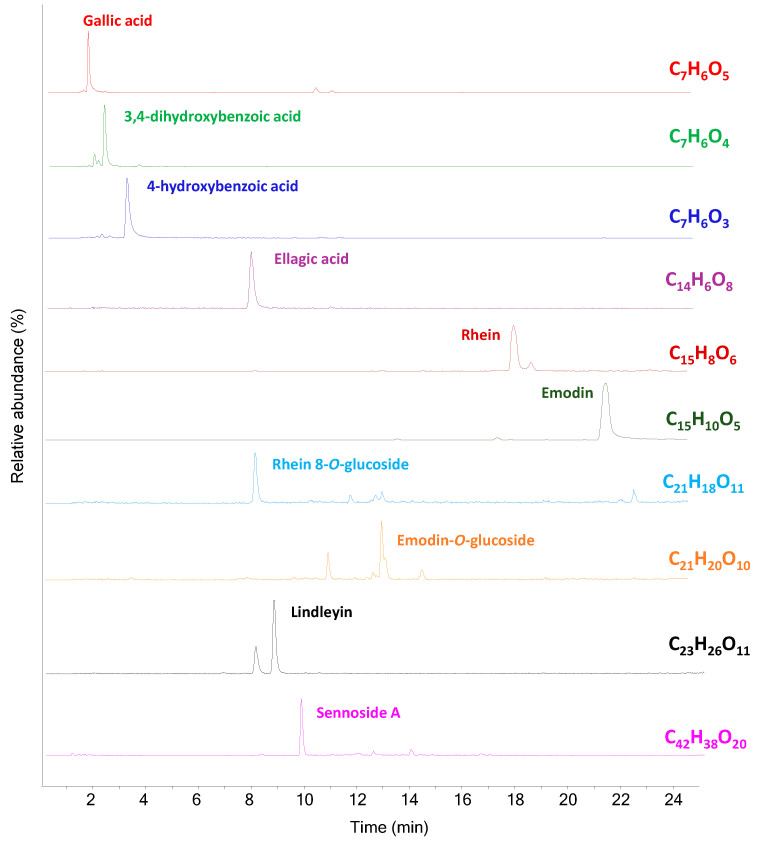
EIC profiles obtained from HPLC–MS/MS analysis of the extract of sample IF 993 (negative mode).

The GC–MS analysis of ointments IF 993, IF 698, IF 323, IF 1317 and IF 2407 (Figures S14–S18) did not show any known marker ascribable to a specific material. Nevertheless, the signal of rhein (#122) was detected in the GC–MS chromatogram of the acidic fraction of sample IF 993 (Figure S14). This ointment was unlabeled; thus, no hypothesis on the formulation could be possible. Rhein is an anthraquinone that can be extracted from rhubarb species and is known to have medicinal properties [31,32]. Sample IF 993 was analyzed by HPLC–MS/MS to detect the presence of organic polar compounds. The HPLC chromatographic profile of the sample shows numerous peaks mainly belonging to tannins and anthraquinones, many of which are present in their glycosylated forms. Figure 6 shows the EICs of the compounds identified. Rhein, emodin, lindleyin and sennosides are reported to be the constituents of the plants of Rheum species, which include rhubarb, thus further confirming the hypothesis formulated on the basis of the GC–MS analysis [33,34].

Samples IF 698 and IF 1317 were analyzed by Py–GC–MS and the resulting pyrograms are reported in Figure S19 of the Supplementary Materials. Both the samples present the signals of anhydrosugars and other products formed from the pyrolysis of carbohydrates [35]. This means that both the ointments contain saccharide or polysaccharide materials. Sample IF 1317 is labelled as “Alsidium helminthochorton thallus”, an alga having anthelmintic properties. Algae are mainly composed of cellulose and hemicellulose, thus confirming the correspondence between the results obtained by analytical pyrolysis and the label of the jar [36].

A similar result was obtained for sample IF 698. Also in this case, the presence of a saccharide or polysaccharide material is consistent with the label reported on the jar, as the ointment is supposed to be made of pulp of elderberry fruits, mainly composed of sugars and polysaccharides [37].

## 3. Materials and Methods

### 3.1. Chemicals

All chemicals were purchased from Sigma Aldrich (Milan, Italy) and were used without any further purification. Acetonitrile, water, chloroform, diethyl ether, ethanol, n-hexane, isopropanol and methanol were of HPLC or LC-MS grade. Tridecanoic acid, hexadecane (internal standards), N,O-bis(trimethylsilyl)trifluoroacetamide (BSTFA) and hexamethyldisilazane (HMDS) (derivatization agents) were purchased from Sigma-Aldrich (purity > 99%). The other reagents used, potassium hydroxide, hydrochloric acid and isooctane, were of analytical quality.

### 3.2. Samples

Fourteen containers belonging to the collection were selected for collecting samples to be chemically characterized. Eight of the samples (IF 1908, IF 1909, IF 1918, IF 1906, IF 1914, IF 1916, IF 2407, IF 2405) were collected from containers in the 18th-century medicine chest mentioned in the Introduction. Some of those still presented labels indicating their complex formulations, while a few were devoid of any label. The container numbered IF 1916 was particularly intriguing, due to the complexity of the historical recipe reported for the Pulv. Bezoard Sennert (as the content is described on the label) and the heterogeneous appearance of the content of the glass jar, observed under an optical microscope. In addition, other samples were collected from containers belonging to the collection as separate lots gathered from other pharmacies in Transylvania, dated between the 18th and the early 20th century. It is of specific interest to gather information on recipes of the famous Panacea drug during this time period, such as mummy powder and theriac (as mentioned on the labels of IF 341, IF 323, IF 1857), since their increased use in Transylvania during the 18th century and persistence into the modern period seems to be a typical regional development [3]. Two additional 18th-century containers were selected: one since the label mentions a locally available organic ingredient (IF 698—elderberry fruit pulp) and the other for the exotic content (IF 1317—Corsican moss). The content of IF 993 required identifications as the lozenge-shaped inscription field on the label is empty.

Table 4 lists the samples investigated in this work along with a description of the containers.

Sampling was performed paying strict attention to avoid any contamination. Objects were handled with nitrile gloves, clean scalpels were used, and the sampled contents were wrapped in new aluminum foil until analysis. The most challenging sampling was the extraction of material from jar IF 2405, since the corroded cap of the vessel could not be removed. Any attempt to access the content from above would have led on one hand to the contamination of the content, and on the other hand would have seriously compromised the value of the jar as part of an exposition in the museum collection. Consequently, it was decided to reach the content of jar IF 2405 by drilling a hole in the bottom of the glass container, assuming that the powder resulting from the glass would not interfere with the following analyses of the content.

### 3.3. GC–MS Analysis

About 1 mg of each sample was analyzed by using a procedure optimized for the identification of lipid and resinous organic materials in archaeological samples [18]. In detail, samples were subjected to alkaline hydrolysis by adding 1 mL of hydroalcoholic solution of KOH (KOH in CH_3_OH (10% weight)/KOH in H_2_O (10% weight), 2:3). Neutral and acid organic components were extracted in two separate fractions with *n*-hexane and—after acidification with HCl—with diethyl ether, respectively. The fractions obtained were separately subjected to the derivatization reaction with BSTFA and 2 μL of the obtained solutions were analyzed by GC–MS. Analyses were performed using a 6890 N GC system gas chromatograph (Agilent Technologies, Palo Alto, CA, USA) coupled with a 5975 mass selective detector (Agilent Technologies) single-quadrupole mass spectrometer equipped with a PTV injector. The mass spectrometer was operated in the EI positive mode (70 eV) analyzing mass-to-charge values in the range *m*/*z* 50–700. For chromatographic separation, an HP-5MS fused silica capillary column (5% diphenyl/95% dimethyl-polysiloxane, 30 m × 0.25 mm i.d., 0.25 μm film thickness, J&W Scientific Agilent Technologies, Folsom, CA, USA) with a deactivated silica precolumn (2 m × 0.32 mm i.d., J&W Scientific Agilent Technologies) was used. Chromatographic conditions: initial temperature 80 °C, 2 min isothermal, 10 °C/min up to 200 °C, isothermal 3 min, 10 °C/min up to 280 °C, isothermal 3 min, 20 °C/min up to 300 °C, isothermal 20 min.

### 3.4. SPME–GC–MS Analysis

About 40 mg of each sample was homogeneously spread on the bottom of a 10 mL glass vial. The vial was sealed with a PTFE/silicone septum. Solid-phase microextraction was performed after 30 min of stabilization at 60 °C using a Stable Flex Divinylbenzene/Carboxen/PDMS fiber (Supelco, Merk, Milan, Italy). The fiber was exposed for 30 min at constant laboratory temperature. The analytes were desorbed in a GC–MS injector in splitless mode at 250 °C. The analyses were carried out using the GC–MS system described in the previous section.

Chromatographic conditions: initial temperature 40 °C, 1 min isothermal, 5 °C/min up to 250 °C, isothermal 20 min; transfer capillary temperature 280 °C; ion source 250 °C; injection temperature 250 °C; injection operating with a split flow of 50 mL/min and a splitless time of 0.6 min; electron ionization energy 70 eV; mass-to-charge range 50–1000 *m/z* for GC–MS screening, or 35–700 *m*/*z* for SPME–GC–MS. Helium (purity 99.9995%) was used as carrier gas at 1.2 mL min^−1^ flow rate.

### 3.5. HPLC/MS Analysis of Acyl-Lipids and Waxes

The samples underwent microwave assisted extraction with 400 μL of extracting solution chloroform-hexane (3:2 *v*/*v*), using an Ethos One microwave oven (Milestone, Milan, Italy) working at 600 W, 80 °C for 25 min.

An HPLC 1200 Infinity, coupled to a Jet Stream ESI-Q-ToF 6530 Infinity detector and equipped with an Agilent Infinity autosampler (Agilent Technologies, Palo Alto, CA, USA) and a Jet Stream ESI source, was used for the analysis of the extracts. The ESI source operated in positive ionization mode and the working conditions were: drying gas N_2_ (purity > 98%) temperature 350 °C and 10 L/min flow; capillary voltage 4.5 KV; nebulizer gas pressure 35 psig; sheath gas temperature 375 °C and 11 L/min flow; fragmentor voltage 200 V, nozzle voltage 1000 V, skimmer 65 V octapole frequency 750 V. High resolution MS and MS/MS spectra were acquired in positive mode in the range 100–3200 *m*/*z* (CID voltage 50 V, collision gas N_2_, purity 99.999%).

The chromatographic separation was performed on an analytical reversed-phase column Poroshell 120 EC-C18 (3.0 × 75 mm, particle size 2.7 μm), with a pre-column Zorbax Ecipse Plus C-18 (4.6 × 12.5 mm, particle size 5 μm), both from Agilent Technologies. The eluents were A: methanol/water (85:15) and B: isopropanol. The flow rate was 0.3 mL/min and the program was: 90% A for 5 min, then a linear gradient up to 90% B in 50 min and then hold for 10 min. During the separation, the column was thermostated at 45 °C.

### 3.6. HPLC/MS Analysis of Low Molecular Weight Polar Compounds

The samples were extracted by sonication using 200 μL of extracting solution 0.1% Na_2_EDTA in H_2_O/DMF (1:1, *v*/*v*) at 60 °C for 60 min, as previously optimized for organic dyes and pigments, including tannins. The extracts were filtered with PTFE syringe filters (0.45 μm) and injected in the HPLC–MS/MS system.

An HPLC 1200 Infinity, coupled to a Jet Stream ESI-Q-ToF 6530 Infinity detector and equipped with an Agilent Infinity autosampler (Agilent Technologies), was used. The mass spectrometer operated in ESI ionization in negative mode and the working conditions were: drying gas N_2_ (purity > 98%) temperature 350 °C and 10 L/min flow; capillary voltage 4.5 KV; nebulizer gas pressure 35 psi; sheath gas temperature 375 °C and 11 L/min flow; fragmentor voltage 175 V. High resolution MS and MS/MS spectra were acquired in negative mode in the range 100–1700 m/z at a scan rate of 1.04 spectra/sec (CID voltage 30 V, collision gas N_2_, purity 99.999%).

The chromatographic separation was performed on a Poroshell 120 EC-C18 reversed-phase analytical column (3.0 × 75 mm, particle size 2.7 μm), with a Zorbax pre-column (4.6 × 12.5 mm, particle size 5 μm), both Agilent Technologies. The eluents were A: formic acid (FA 0.1% *v*/*v*) in LC-MS grade water and B: formic acid (FA 0.1% *v*/*v*) in LC-MS grade acetonitrile. The flow rate was 0.4 mL/min and the program was: 5% B for 2.6 min, then to 50% B in 13.0 min, to 70% B in 5.2 min, to 100% B in 6.2 min and then hold for 8.0 min; re-equilibration took 11 min. During the separation, the column was thermostated at 30 °C.

### 3.7. Py–GC–MS Analysis

Py–GC–MS measurements were performed with an EGA/PY-3030D micro-furnace pyrolyzer (Frontier Laboratories) coupled to a 6890 gas chromatograph equipped with a split/splitless injector, with a slip ratio of 10:1, and a 5973 mass spectrometric detector (Agilent Technologies) single-quadrupole mass spectrometer. All experiments were carried out at pyrolysis temperature 550 °C, keeping the interface between the furnace and the GC injector at 280 °C. The mass spectrometer was operated in the EI positive mode (70 eV) analyzing mass in the range *m*/*z* 50–600. For the chromatographic separation, an HP-5MS fused silica capillary column (5% diphenyl/95% dimethyl-polysiloxane, 30 m × 0.25 mm i.d., 0.25 μm film thickness, J&W Scientific Agilent Technologies with a deactivated silica precolumn (2 m × 0.32 mm i.d., J&W Scientific Agilent Technologies) was used. Peak assignment was based on the comparison with the literature and libraries of mass spectra (NIST 20 main EI MS library). Approximately 100 µg of sample was used for each analysis, adding 2 µL of HMDS as a derivatizing agent.

### 3.8. Field Emission Gun–Scanning Electron Microscopy (FEG–SEM)

A FEI Quanta 450 ESEM FEG environmental field emission instrument (ESEM, FEI Company, Eindhoven, the Netherlands) equipped with an energy-dispersive spectrometer (EDS) for X-ray microanalysis (Bruker Nano GmbH, Bruker, Billerica, MA, USA) with a QUANTAX XFlash Detector 6|10. Analyses were performed under high vacuum and at a fixed working distance of 10.0 mm with an electron beam accelerating voltage of 20 kV.

## 4. Conclusions

The data presented above, provided through a multi-analytical approach based on different analytical techniques, are the first analytical results characterizing the organic materials in 14 historical pharmaceutical ointments of the valuable History of Pharmacy Collection of the National Museum of Transylvanian History (Cluj-Napoca), thus significantly contributing to the knowledge on the collection. Furthermore, the analytical data complement the scarce information regarding the *materia medica* in Eastern European collections.

Twelve of the fourteen studied mixtures contained analytically interpretable organic content, in some cases showing interesting correspondence with the label reported on the vessels. Analyses revealed the content of the unlabeled jar IF 993 as rhubarb extract, a healing material still used today for digestive problems, while the other jar with unidentified content, IF 1918, contained a (poly-)saccharide material and possibly a relatively well-preserved vegetable oil. Jars IF 2407 and IF 323, featuring a label difficult to be interpreted, did not show to contain relevant organic constituents. The determined content of jars IF 698 and IF 1317 matches with the ingredient reported on the label, while the composition of the ointments IF 1908, IF 1909 and IF 1916 only partially align with the ingredients list. The composition of ointments IF 1906, IF1914, IF 341, IF 1857 and IF 2405 proved different from those expected on the bases of the labels, and chemical analysis highlighted a discrepancy among the actual ingredients and those reported in the historical label. The collection had indeed a very discontinuous conservation history.

In the case of jar IF 1906, the detection of lead in the amorphous material is consistent with the hypothesized formulation of the ointment.

Finally, the presence of pentachlorophenol in some samples is a sign of the fact that the collection suffered from a mold infestation that was treated in the past with this antifungal agent.

The present analyses revealed the chemical compositions of most of the jars and highlighted that during the second half of the 18th century the Transylvanian upper classes had access to expensive and rare drugs and were following the era’s general trends of using both traditional remedies and new chemical drugs.

## Figures and Tables

**Table 1:** Summary of the techniques applied (✓), and materials identified in the samples investigated.

Ointment	GC–MS	SPME–GC–MS	HLPC-MS	PY–GC–MS	FEG–SEM	Summary
IF 1908	✓	✓	✓			Vegetable oil; animal fat; beeswax; pine resin; aromatic herbs
IF 1909	✓	✓				Jalap root; pine resin
IF 1918	✓		✓	✓		Vegetable oil (not confirmed); polysaccharide material
IF 1906	✓	✓	✓		✓	Animal fat; pine resin; lead
IF 1914	✓					Vegetable oil or animal fat
IF 1916	✓	✓	✓		✓	Pine resin; animal origin material; tannin-based material; calcium; iron; aluminum; zirconium; gold; lead; mercury; antimony
IF 2407	✓	✓		✓		-
IF 2405	✓	✓	✓			Pine resin (from *Pinus sylvestris* L.)
IF 341	✓	✓	✓			Mastic resin
IF 698	✓	✓		✓		Elderberry fruit
IF 323	✓	✓		✓		-
IF 993	✓		✓			Rhubarb extract
IF 1857	✓	✓		✓		Pine resin
IF 1317	✓	✓	✓	✓		*Alsidium helminthochorton* (Schwendimann) Kütz. seaweed

**Table 2:** List of the compounds identified in the samples investigated. Base peaks in the mass spectra are in bold. Some of the compounds were detected as trimethylsilyl derivatives (TMSs).

#	Compounds	Retention Time (min)	Main Ions (*m*/*z*)
1	Oxalic acid	6.2	**73**, 147, 190, 219
2	2-decanone	7.2	**58**, 71, 156
3	Borneol	7.6	**95**, 108, 121, 136, 226
4	*cis*-verbenol	7.6	73, **144**, 209, 224
5	Myrtenol	8.5	73, **91**, 103, 165, 224
6	*cis*-carveol	8.7	**73**, 119, 141, 181, 209, 224
7	Succinic acid	8.8	73, **147**, 247
8	Glutaric acid	10.0	73, **147**, 261
9	Longifolene	10.1	**91**, 105, 161, 189, 204
10	Myrtenoic acid	10.4	**73**, 105, 179, 223
11	Decanoic acid	10.6	73, 75, **117**, 129, 229, 244
12	Malic acid	11.1	**73**, 147, 233, 245, 335
13	4′-hydroxyacetophenone	11.2	**193**, 208
14	Adipic acid	11.2	**73**, 111, 147, 275
15	Citric acid	11.7	**73**, 147, 201, 375
16	Cinnamic acid	11.7	75, 103, 131, 161, **205**, 220
17	4-hydroxypentenoic acid	12.0	75, 117, 129, **169**, 245
18	Kojic acid	12.0	**73**, 147, 271
19	Hydroxyglutaric acid	12.2	73, **129**, 147, 247, 349
20	Hexadecane (IS1)	12.3	**57**, 71, 85, 226
21	Pimelic acid	12.4	**73**, 125, 147, 173, 289
22	8-hydroxyoctadecanoic acid	12.6	75, **147**, 199, 289
23	7-hydroxynonanoic acid	12.7	**73**, 131, 217, 289, 303
24	4-hydroxybenzoic acid	12.7	**73**, 193, 223, 267, 282
25	4-hydroxy-3-methoxybenzoic acid, methyl ester	12.8	123, **151**, 182
26	Dodecanoic acid	12.9	73, **117**, 129, 145, 257, 272
27	Tartaric acid	13.0	**73**, 147, 219, 292, 423
28	4-hydroxyhexenoic acid	13.2	75, 117, 129, **259**
29	Suberic acid	13.5	**73**, 129, 147, 187, 303
30	8-hydroxydecanoic acid	13.5	**73**, 131, 217, 303, 317
31	Phthalic acid	13.5	73, **147**, 295, 310
32	Tridecanoic acid (IS2)	14.0	73, **117**, 129, 145, 271, 286
33	Aconitic acid	14.1	73, **147**, 229, 375
34	Tetradecanol	14.1	75, 103, **271**
35	4-hydroxyhydrocinnamic acid	14.2	73, **179**, 192, 310
36	Vanillic acid	14.3	**73**, 223, 267, 282, 297, 312
37	9-hydroxyundecanoic acid	14.4	**73**, 131, 217, 317, 331
38	Terephthalic acid	14.5	103, 221, **295**, 310
39	Azelaic acid	14.5	**73**, 129, 147, 201, 317
40	Citric acid	15.1	73, 147, **273**, 347, 363, 465
41	2-(4-hydroxyphenyl)-2-oxoacetic acid	15.1	73, **193**, 295, 310
42	4-methoxycinnamic acid	15.1	**161**, 191, 235, 250
43	Methyl salicylate	15.1	92, **120**, 152
44	Myristic acid	15.1	73, **117**, 129, 145, 285, 300
45	Pentadecanol	15.3	75, 103, **285**
46	Phthalate	15.5	**149**
47	Pentachlorophenol	15.6	73, 93, **323**, 338
48	Sebacic acid	15.9	**73**, 129, 147, 215, 331
49	Syringic acid	16.1	73, 297, 312, **327**, 342
50	Methyl palmitate	16.2	**74**, 87, 227, 270
51	Ferulic acid	16.4	73, 249, 308, 323, **338**
52	Pentadecanoic acid	16.6	73, **117**, 129, 145, 299, 314
53	*p*-coumaric acid	16.6	**73**, 219, 249, 293, 308
54	Hexadecanol	16.8	75, 103, **299**
55	2-hyrdoxyazelaic acid	17.0	73, 147, **303**, 405
56	Undecanedioic acid	17.5	**73**, 129, 147, 229, 345
57	Hexadecenoic acid	17.9	73, **117**, 129, 145, 311, 326
58	8,13-epoxy-labd-14-ene	18.1	81, 95, 137, 177, 192, **257**, 275
59	Palmitic acid	18.3	73, **117**, 129, 145, 313, 328
60	Heptadecanol	18.5	75, 103, **313**
61	3,5-dibromo-2,4-dihydroxybenzaldehyde	18.6	**73**, 425, 438
62	2-hydroxysebacic acid	18.6	73, **317**, 391, 419
63	Isoferulic acid	19.1	73, 249, 308, 323, **338**
64	Gallic acid	17.1	**73**, 281, 443, 458
65	Methyl steareate	19.5	**74**, 87, 255, 298
66	Octadec-9-enol	19.5	75, 103, **325**
67	Heptadecanoic acid	19.7	73, **117**, 129, 145, 327, 342
68	Octadecanol	19.9	75, 103, **327**
69	Methyl 15-hydroxyhexadecanoate	20.5	73, **117**, 311, 343, 358
70	Linoleic acid	20.6	**73**, 117, 129, 145, 337, 352
71	*trans*-Oleic acid	20.6	**73**, 117, 129, 145, 339, 354
72	*cis*-Oleic acid	20.8	**73**, 117, 129, 145, 339, 354
73	18-hydroxykaur-16-ene	20.8	57, 73, **257**, 270, 345, 360
74	Stearic acid	20.9	73, **117**, 129, 145, 341, 356
75	Nonadecanol	21.1	75, 103, **341**
76	Levopimaric acid	21.2	**73**, 121, 141, 159, 359, 374
77	Isopimaric acid	21.5	**73**, 241, 256, 359, 374
78	Tricosane	21.5	**57**, 71, 85, 324
79	Dehydroabietinol	21.8	173, 239, **253**, 343, 358
80	14-hydroxyhexadecanoic acid	21.8	73, **131**, 311, 387, 401
81	15-hydroxyhexadecanoic acid	21.8	73, **117**, 217, 311, 385, 401
82	Cryptopimaric acid	21.8	73, **121**, 241, 257, 359, 374
83	Pimaric acid	21.9	**73**, 121, 241, 257, 359, 374
84	Sandaracopimaric acid	22.1	73, **121**, 241, 257, 359, 374
85	Eicosanol	22.1	75, 103, **355**
86	Methyl dehydroabietate	22.2	239, 299, 314
87	Didehydroabietic acid	22.4	73, **237**, 252, 355, 370
88	Tetracosane	22.6	**57**, 71, 85, 338
89	Dehydroabietic acid	22.7	73, **239**, 357, 372
90	Abietic acid	22.9	73, 241, **256**, 359, 374
91	Eicosanoic acid	23.0	73, **117**, 129, 145, 369, 384
92	Methyl 9,10-dihydroxystearate	23.2	73, 215, **259**, 332
93	Methyl 7-β-hydroxydehydroabietate	23.4	73, 176, **237**, 285, 387, 402
94	Pentacosane	23.5	**57**, 71, 85, 352
95	Methyl 7-methoxydehydroabietate	23.7	176, **237**, 253, 312, 344
96	7β-hydroxydehydroabietic acid	23.7	73, **191**, 237, 417, 460
97	Docosanol	24.0	75, 103, **383**
98	*Erythro*-9,10-dihydroxyoctadecanoic acid	24.0	73, 129, 147, 215, **317**, 390
99	Methyl 15-hydroxydehydroabietate	24.1	73, 237, 269, **387**, 402
100	*Threo*-9,10-dihydroxyoctadecanoic acid	24.1	73, 129, 147, 215, **317**, 390
101	10,16-dihydroxyhexadecanoic acid	24.2	73, 129, 147, 303, 317, **331**, 489, 504
102	8,16-dihydroxyhexadecanoic acid	24.2	73, 129, 147, **303**, 489
103	Hexacosane	24.3	**57**, 71, 85, 366
104	1-monopalmitin	24.4	73, 129, 147, **371**, 459
105	9,18-dihydroxyoctadecanoic acid	24.4	73, 215, **317**, 390, 517
106	Methyl 7-oxodehydroabietate	24.4	**253**, 313, 328
107	7-oxodehydroabietic acid	24.5	73, 187, **253**, 268, 371, 386
108	Docosanoic acic	24.7	73, **117**, 129, 145, 397, 412
109	Batyl alcohol	25.1	57, 73, 117, 147, **205**, 341
110	Heptacosane	25.2	**57**, 71, 85, 380
111	3,6,2′,3′-tetramethoxyflavone	25.3	151, **311**, 327, 342
112	Tetracosanol	25.6	75, 103, **411**
113	Methyl 15-hydroxy-7-oxodehydroabietate	25.7	73, 286, **401**
114	1-monostearin	25.9	73, 147, **399**, 487
115	Octacosane	25.9	**57**, 71, 85, 394
116	15-hydroxy-7-oxodehydroabietic acid	26.0	73, 285, 341, **459**
117	Tetracosanoic acid	26.2	73, **117**, 129, 145, 425, 440
118	Nonacosane	26.6	**57**, 71, 85, 408
119	Hexacosanol	27.0	75, 103, **439**
120	Triacontane	27.4	**57**, 71, 85, 422
121	1,23-tetracosandiol	27.5	73, **117**, 149, 191
122	Rhein	27.6	45, 73, **485**
123	Hentriacontane	28.1	**57**, 71, 85, 436
124	Octacosanol	28.5	75, 103, **467**
125	Cholesterol	28.8	73, **129**, 329, 353, 368, 458
126	Dotriacontane	29.0	**57**, 71, 85, 450
127	1,25-hexacosandiol	29.1	73, **117**, 149, 191
128	1-tritriacontene	29.7	**55**, 69, 83, 462
129	Oleana-11,13(18)-diene	29.8	55, 69, 255, 393, **408**
130	Campesterol	29.9	55, 73, **129**, 343, 367, 382, 457, 472
131	Tritriacontane	30.0	**57**, 71, 85, 464
132	Stigmasterol	30.2	**83**, 129, 255, 379, 394, 469, 484
133	Triacontanol	30.5	75, 103, **495**
134	Ellagic acid	30.5	**73**, 487
135	β-sitosterol	30.9	73, **129**, 357, 381, 396, 471, 486
136	Olean-18-ene	31.0	55, 69, 177, 189, **204**, 218, 395, 410
137	β-amyrone	31.1	189, 203, **218**, 409, 424
138	1,27-octacosandiol	31.3	73, **117**, 149, 191
139	β-amyrin	31.5	189, 203, **218**, 498
140	Lup-20(29)-en-3-one	31.8	95, **109**, 121, 189, 205, 218, 313, 409, 424
141	Unknown triterpene	32.5	**203**, 281, 408
142	Dotriacontanol	33.2	75, 103, **523**
143	Ursa-9(11),12-dien-3-one	34.0	255, 269, 407, **422**
144	20,24-epossi-25-idrossidammaren-3-one	34.5	125, **143**, 161, 205, 399
145	Moronic acid	34.8	73, **189**, 203, 307, 409, 511, 526
146	Betulone	34.9	73, 95, 189, 203, **409**, 422
147	Oleanonic acid	35.2	73, 189, **203**, 408, 511, 526
148	Oleanoinic aldehyde	35.3	95, 109, 189, **203**, 232, 424, 438
149	Betulonic acid	35.4	**73**, 189, 203, 307, 409, 444, 511, 526
150	Oleanolic acid	35.6	73, 189, **203**, 320, 482, 510, 600
151	Masticadienonic acid	38.2	**73**, 95, 169, 421, 511, 526
152	Isomasticadienonic acid	40.3	73, **95**, 169, 421, 511, 526

**Table 3:** Fatty acids percentages for the four samples that featured a significant amount of fatty acids after saponification.

Sample	C12:0	α,ω-C8	α,ω-C9	C14:0	α,ω-C10	C16:0	C18:1	C18:0
IF 1908	0.8	4.2	23.2	9.7	3.4	38.0	4.4	16.3
IF 1918	0.0	0.0	0.8	2.0	0.1	25.4	51.4	20.3
IF 1914	1.0	1.2	5.2	3.8	1.2	35.0	20.9	31.7
IF 1906	0.0	5.8	13.5	3.7	1.4	45.3	8.0	22.3

**Table 4:** List of the vessels and ointments investigated. Possible ingredients and recipes are suggested built upon historical references, based on the label placed on each container.

Sample	Picture	Description
IF 1908	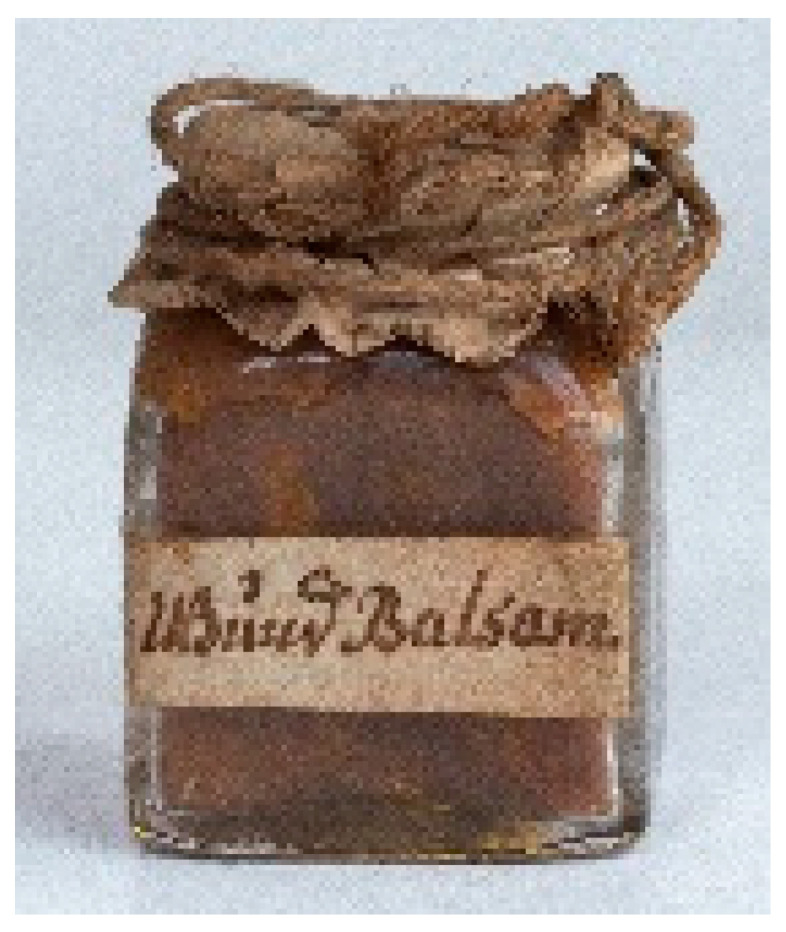	Rectangular glass container with a paper label on one side and a paper cover secured with a thread. Both labels, handwritten in black ink, report: *Wund balsam*. According to recipes dating back to the 18th and early 19th centuries, *Wund Balsam* could include ingredients such as turpentine, myrrh and Galbanum resin, along with various other compounds [38]. Interestingly, the main written sources for Transylvanian *materia medica* do not mention this medicine, and since the label is in German, the recipe might have been borrowed from the German-speaking regions of Europe.
IF 1909	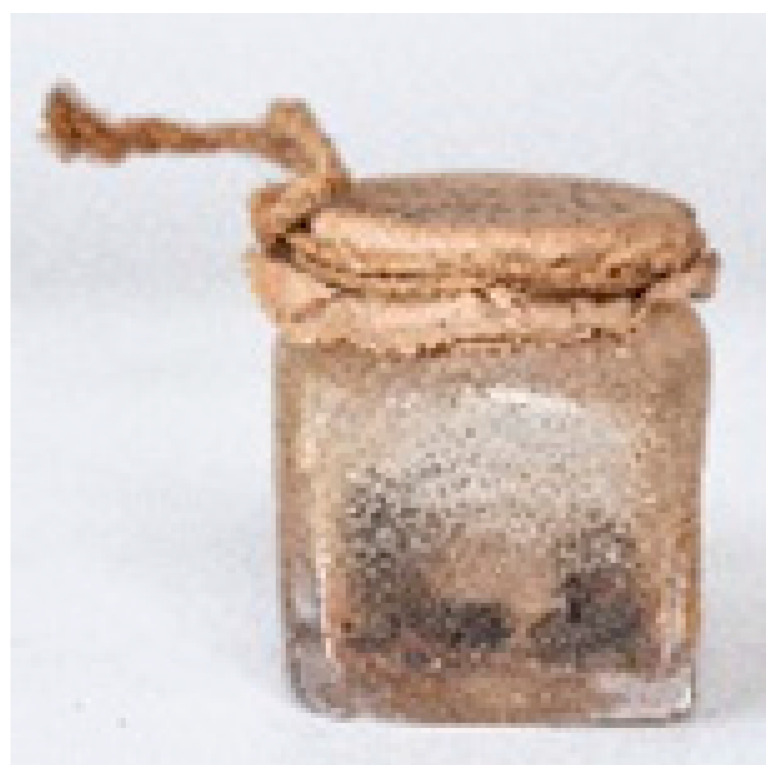	Rectangular glass container with a paper cover tied with a thread, with a handwritten inscription label *Resin Jallap*. This resin was introduced as a medical herb from South America to Europe in the 16th century [23,24]. Besides the resin, this ointment could contain starch, polysaccharidic gums and coloring materials. This formulation was well known in Transylvanian *materia medica* between the 16th and the 19th century [3].
IF 1918	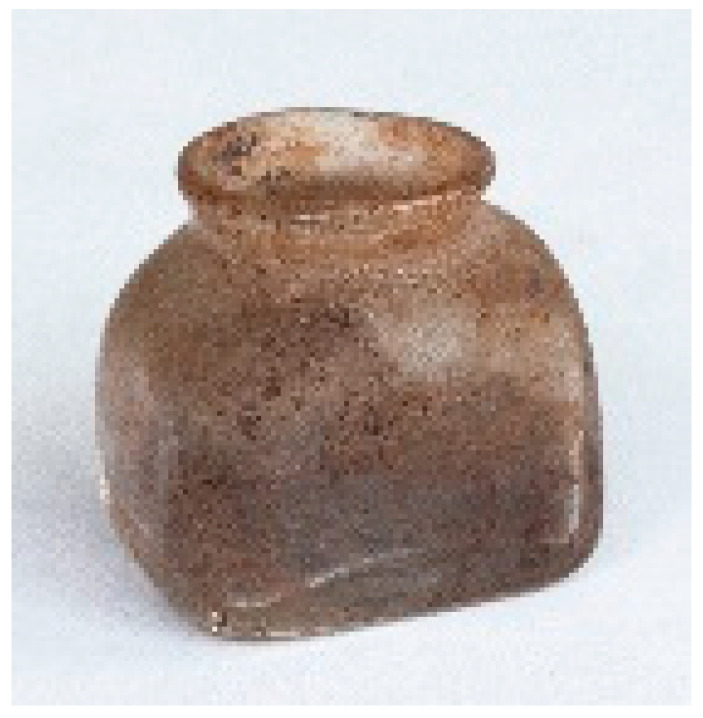	Rectangular glass container missing the paper cover and inscription label. The nature of the preserved ointment is unknown.
IF 1906	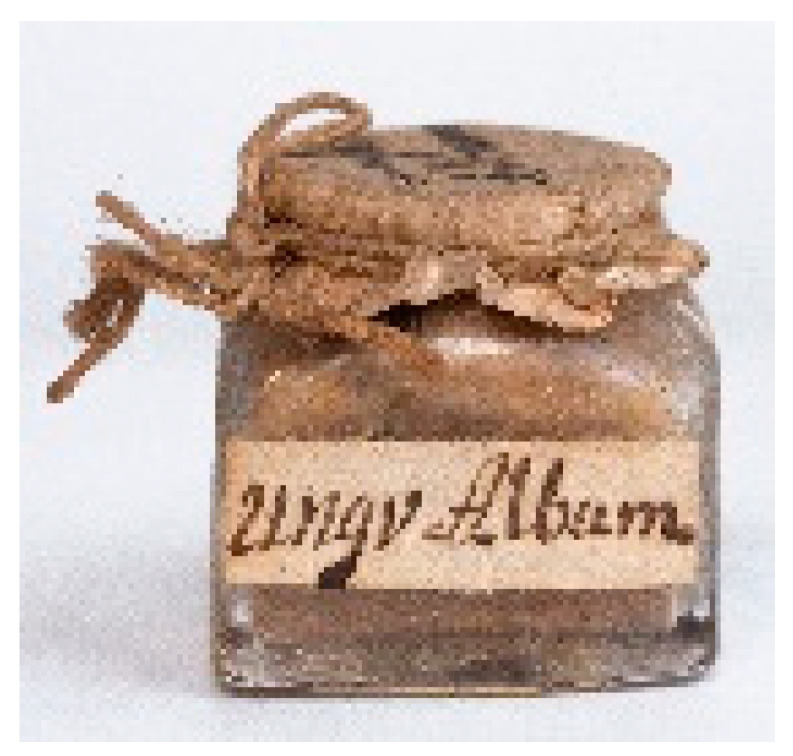	Rectangular glass container with both inscriptions reading *ungv Album*. Based on the historical recipes of the time [38], this could refer to a mixture of olive oil, white wax, and Spermaceti (whale wax), possibly including also lead white. The Austrian dispensatory used in Transylvania mentions the simple variant, with pig lard and Venetian ceruse (lead white) dissolved in vinegar and a variant with camphor [39]. The unguent is mentioned in most of the written sources regarding the era’s *materia medica* in the region [3].
IF 1914	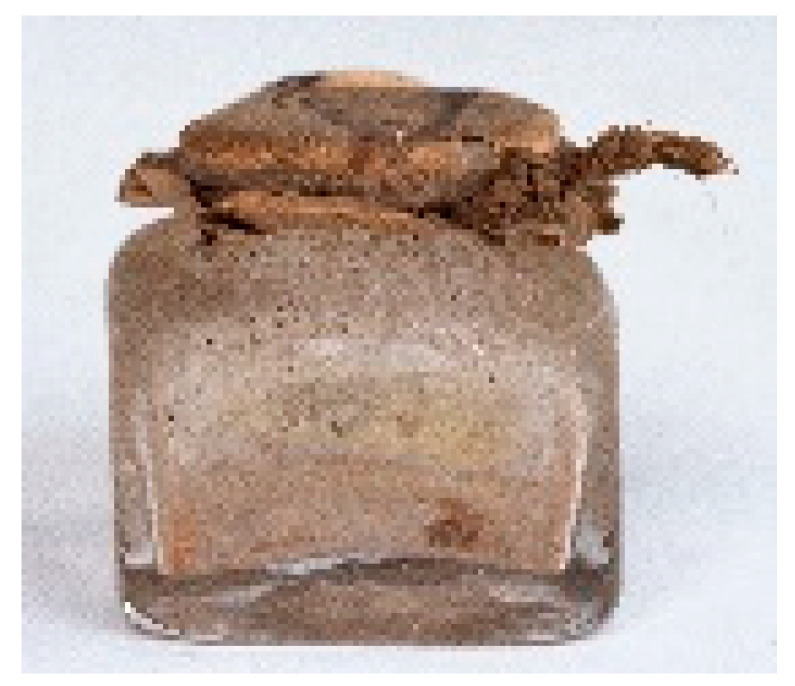	Small rectangular glass container with paper cover tied with a thread with the inscription *gg (compositum): Camphori*. The content was, probably, camphor, used as medication for its scent. Camphor is a constant presence in Transylvanian *materia medica* between the 16th and the 19th century [3].
IF 1916	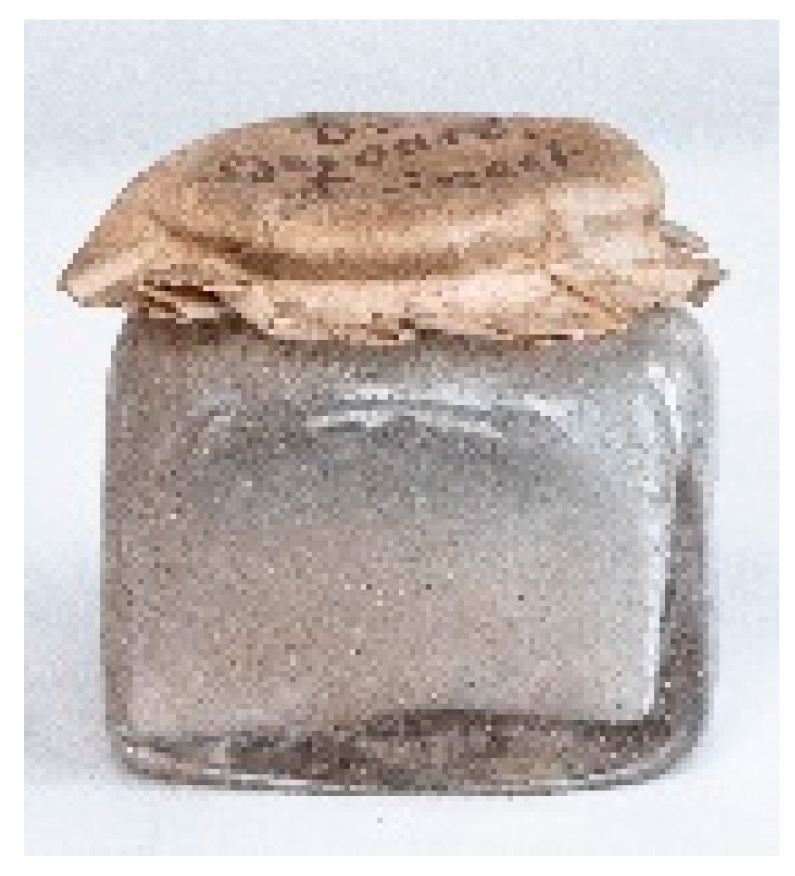	Rectangular glass container with a paper cover, although the thread is missing. The label is handwritten in ink and reads*: [alchemical symbol for Pulvis] Bezoard: Sennert*. Daniel Sennert (1572–1637), a renowned German physician with a keen interest in alchemy and (iatro)chemistry, blended oriental bezoar powder with various other costly ingredients, including deer heart, mother of pearl, red coral, rubies, emeralds, carnelians, jacinth, gold leaf, crabs’ eyes, deer heart bones and terra sigillata. This mixture resulted in a powder with diverse applications, such as in acute diseases, malignant fevers, and epidemics such as small-pox and dysentery [39]. Additionally, this powder served as an ingredient in various other compounded medications. The composite powder is only recorded in the 18th-century pharmacy inventories, price lists and dispensatories from Transylvania [3].
IF 2407	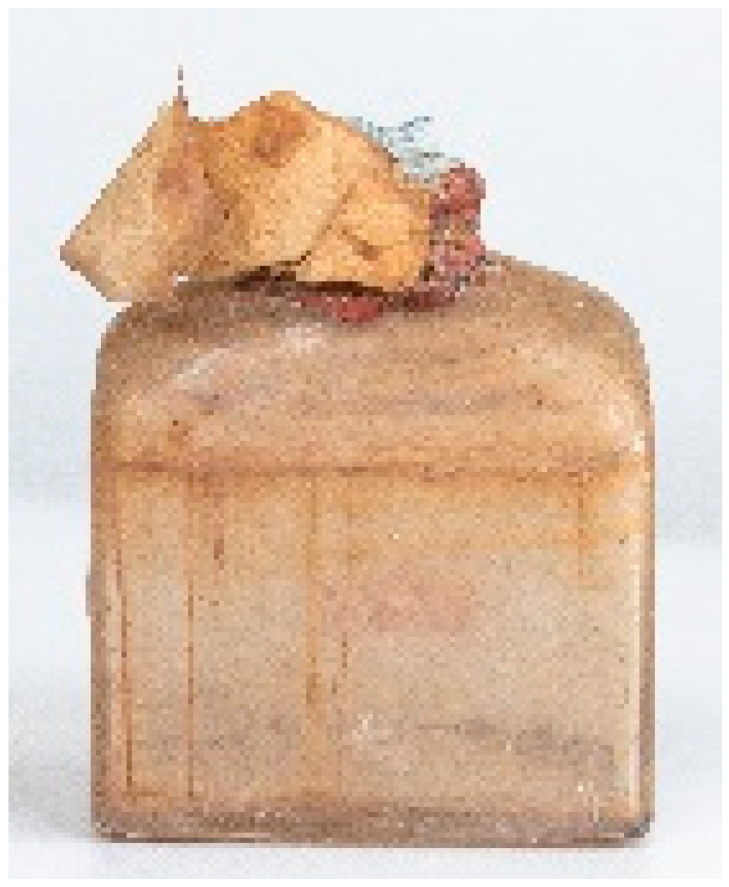	Rectangular clear glass container with a corroded metal screw (cap missing) secured with red wax. A paper inscription label is still attached to the neck with a piece of cord, handwritten in ink, but is rather degraded and burnt/stained. The inscription probably reports the world *Katvolor*. No information on the possible content of this jar is available.
IF 2405	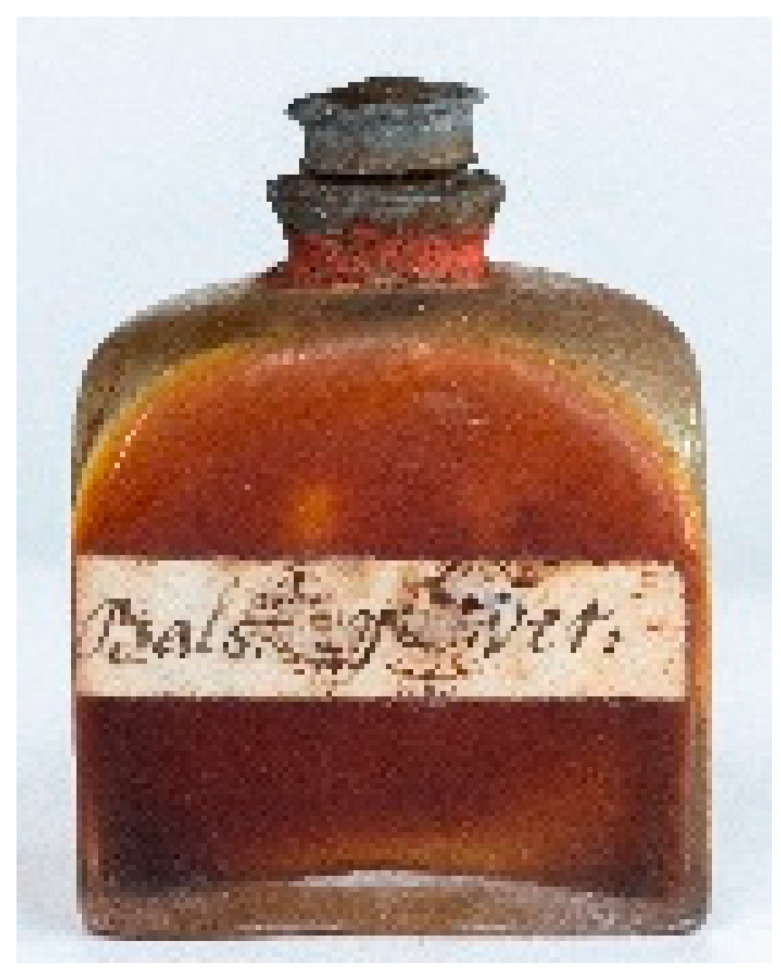	Rectangular glass container with metal screw stopper (the corroded pewter cap of which does not unscrew) secured with red wax, with a paper inscription label on the body, handwritten in ink, probably reporting *Bals pp..ver*. This might originate from “Bals oppo.ver” (Balsamum Oppodeldoc verum), an ointment used for treating wounds. It was described [39] as a blend of olive oil cooked with lithargyrum (crystallized litharge), to which wax, turpentine, laurel oil, as well as several gums and roots were incorporated. The balm is recorded in two 18th-century pharmacy inventories from Transylvania and a couple other written sources [3].
IF 341	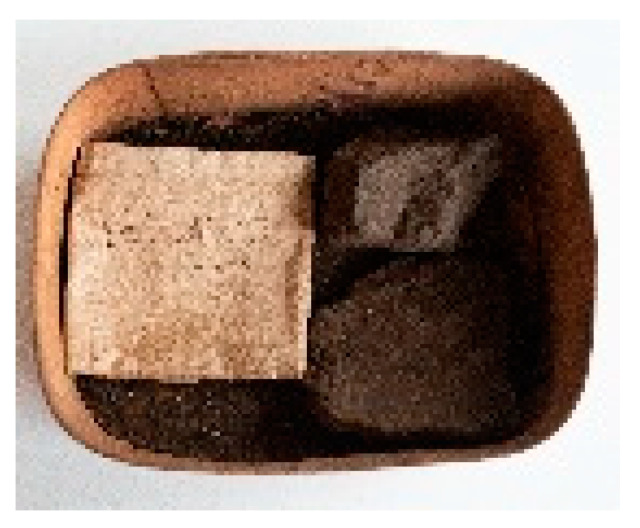	Veneer box containing a paper package with a handwritten inscription, a black powder, and two black solid bodies. The inscription on the package reads *Pulv. Mumiae verae.*, that can be translated into “authentic mummy powder”. The artifact was once used in a pharmacy in Baia Mare, according to the inventory ledger, and can be dated to the 18th–19th century. The pharmacopoeias, inventories, and price lists mention the ingredient consistently between the 16th and the 18th century [3].
IF 698	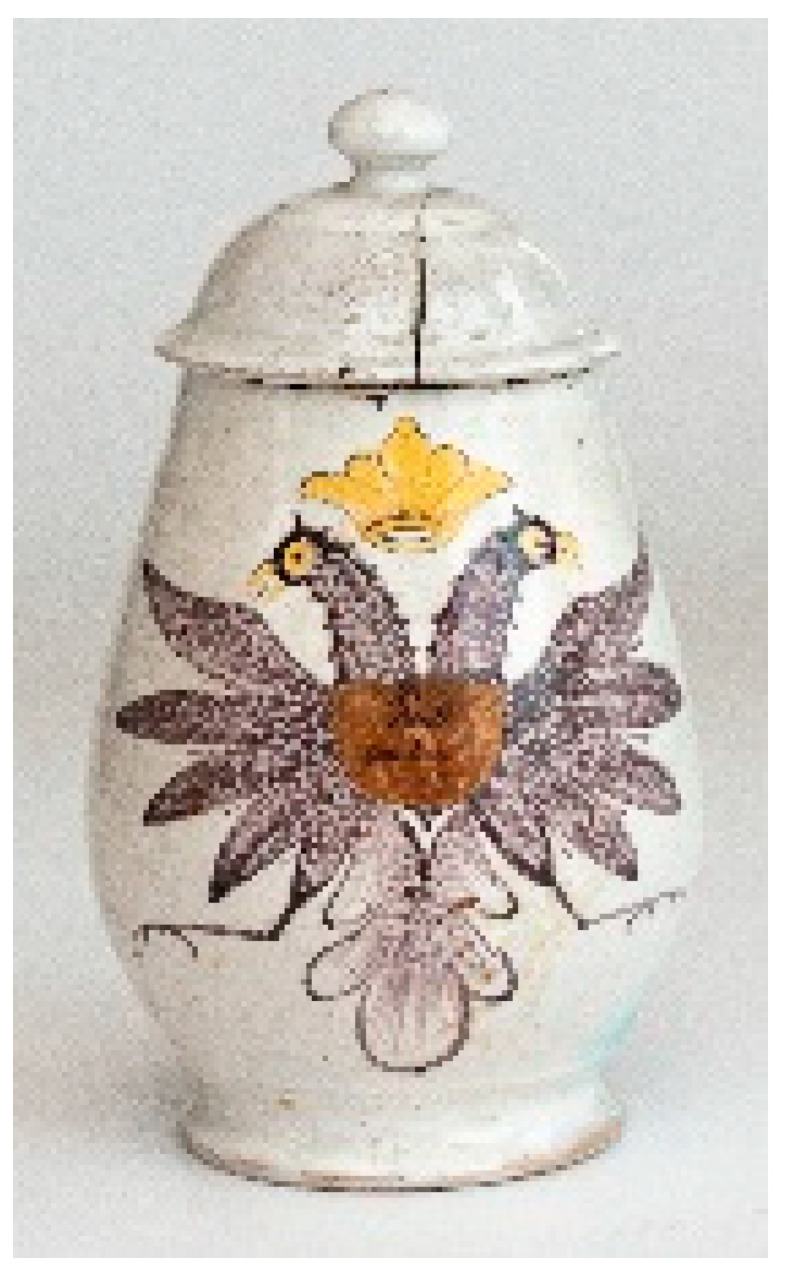	Bellied apothecary jar made of white faience, with domed cover ending in a circular knob. The inscription is written in black cursive letters on a small paper label glued to the body of a two-headed eagle, brown glazed, with a large yellow crown: *Roob sambuci*. This ointment is expected to be made of a concentrated pulp from elderberry fruits, an auxiliary component of medicines for menstruation, urinary issues, calculi and other afflictions [39]. As the museum ledger indicates, it was obtained from the Eagle pharmacy in Mediaș and is dated to the 18th century. Unsurprisingly, as the plant grows in Transylvania, all the pharmaceutical written sources of the early modern and modern eras mention the ointment [3].
IF 323	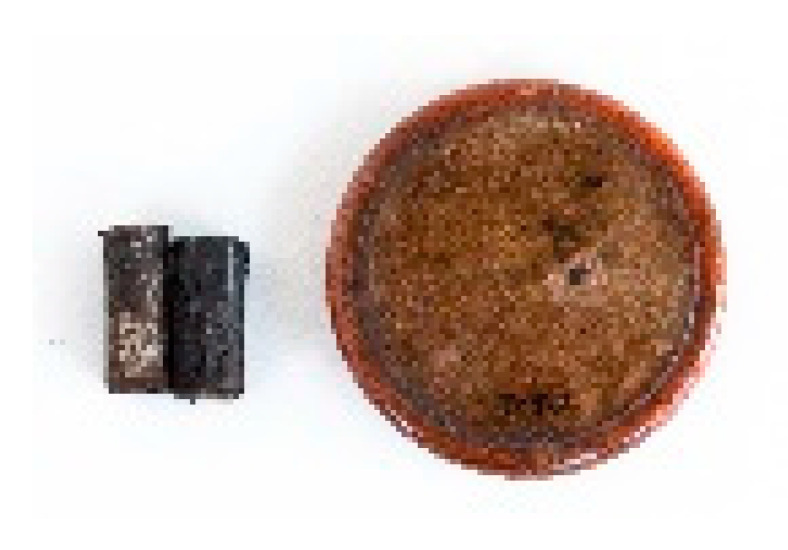	Cylindrical capsules with *materia medica*, one made of metal, with a corroded side, the other made of printed paper, painted black, tied with a thread. The capsules are placed on a turned wood jar cover (likely mismatched, taken from a wooden jar for display purposes). The items, originating from a pharmacy in Baia Mare, can be dated to the 18th–19th century. The content could be *Teriaca veneta,* a popular drug of the period, with a complex organic composition [40].
IF 993	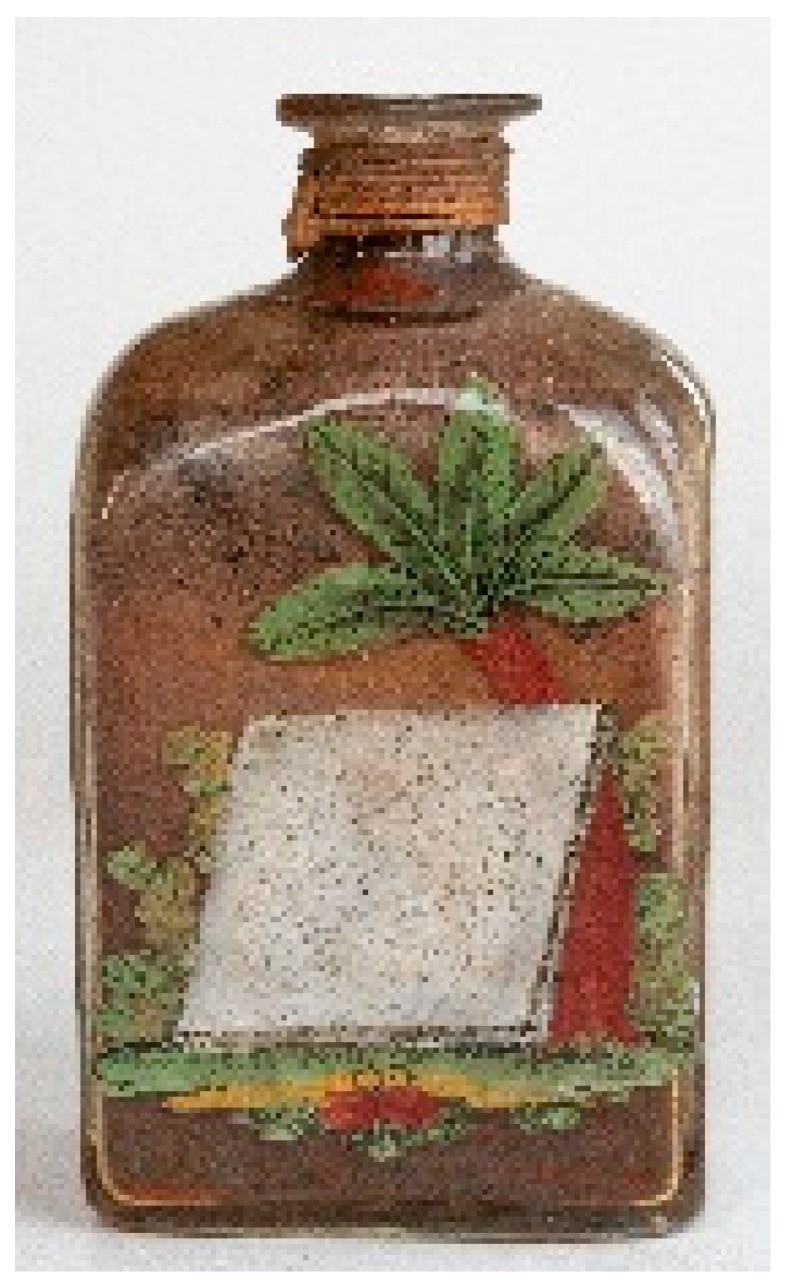	Rectangular jar made of transparent glass, without stopper but with thread coiled around the neck. An entire side is painted with a landscape (tree, grass, bushes) around an empty, lozenge-shaped inscription field with a white background. A small inscription in red cursive can be noted at the base of the neck (N90), and it is likely an old inventory number. According to the museum, the container was obtained from the Golden Lion pharmacy in Orăștie and can be dated to the 18th century.
IF 1857	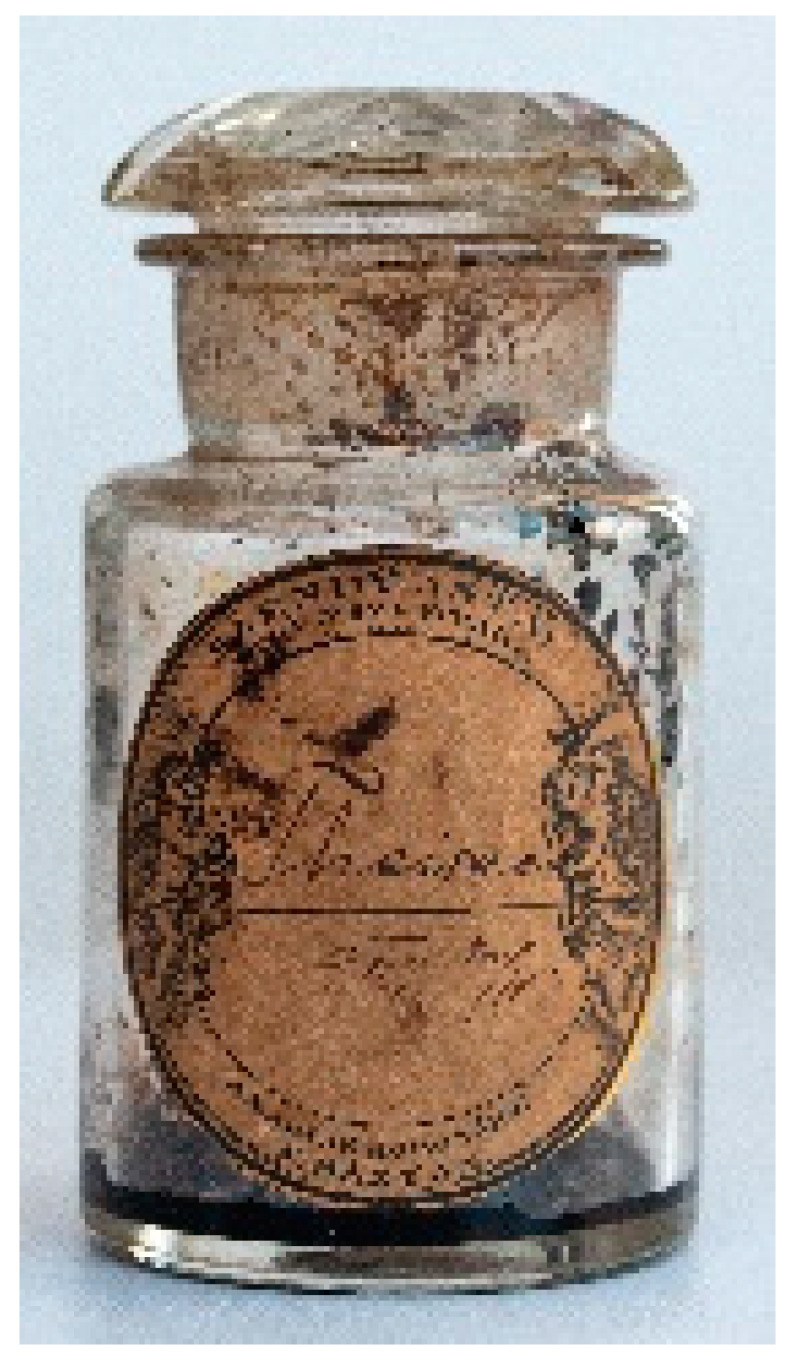	Cylindrical apothecary jar dated to 1907 and made of transparent glass, with a wide neck and an umbrella-shaped faceted glass stopper. The inscription is handwritten in an oval paper label, brown, with the printed emblem of the Hungarian Crown pharmacy in Baia Mare, that includes the depiction of two *puttos* on the sides. The number in red on the neck (4) is likely an old inventory number. The printed inscription of the label is in Hungarian, identifying the pharmacy by name and owner (SZENDŸ ANTAL GYÓGYSZERTÁRA, A MAGYAR KORONÁHOZ N. BÁNYÁN (“Szendy Antal’s the Crown of Hungary pharmacy in Baia Mare”). The handwritten note in the center of the label identifies the content as *Theiraca* (misspelled), the year (“907” with vertical line on top for 1000) and a partially legible information (“..IV”).
IF 1317	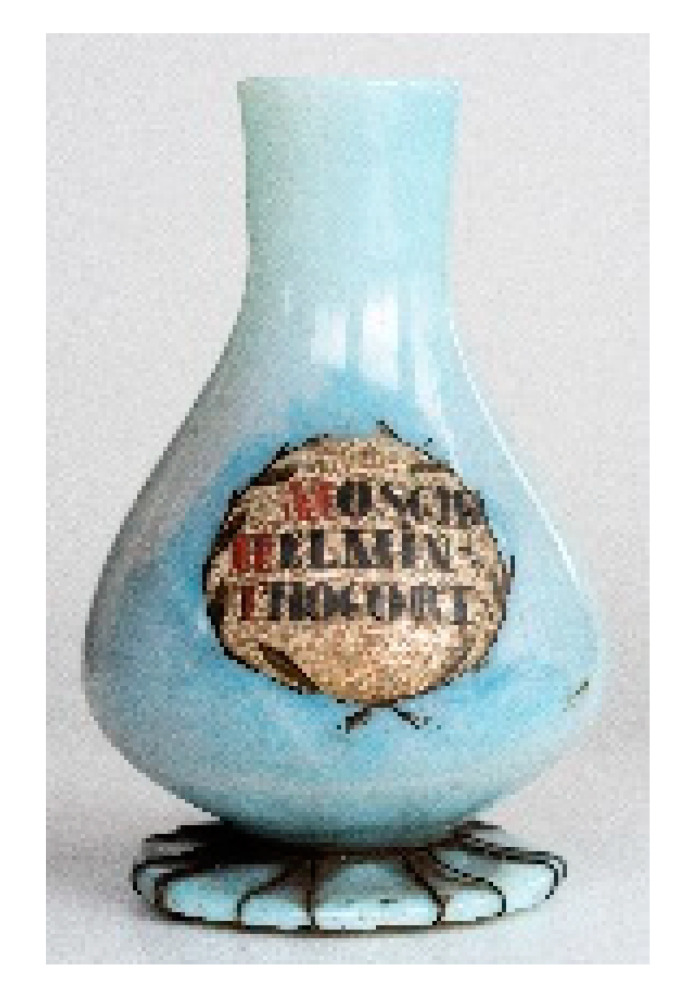	Pear-shaped footed container made of blueish milky glass, with the foot wrapped in a wire net, missing the stopper/cover. The inscription is painted in black capital letters inside a circular cartouche with (mostly faded) vegetal lining consisting of two branches intersecting in the lower part. The inscription reads MOSCH. HELMIN: THOCORT (*Muscus Helminthochorton*) containing Corsican moss (*Alsidium helminthochorton* thallus), used as antiparasitic drug, mostly against intestinal worms [41]. The 18th-century artifact is recorded as once used in a pharmacy from Târnăveni, but contemporary apothecary sources never mentioned the plant in Transylvania.

## Data Availability

The data that support the findings of this study are available from the corresponding author, F.N., upon reasonable request.

## Supplementary Materials

The following supporting information can be downloaded at: https://www.mdpi.com/article/10.3390/molecules29225356/s1, Figure S1: EICs profiles obtained from HPLC–MS/MS analysis of the extract of sample IF 1908. Profiles are obtained by overlapping EICs corresponding to the *m/z* of [M+Na]^+^ reported in literature [17] for the different species. Figure S2: GC–MS chromatograms of (A) acidic fraction and (B) neutral fraction of sample IF 1918. The peaks are labeled according to Table 2. (*): contaminants. Figure S3: Pyrogram of sample IF 1918. TMS: trimethylsilyl derivatives. Figure S4: GC–MS chromatograms of (A) acidic fraction and (B) neutral fraction of sample IF 1914. The peaks are labeled according to Table 2. Figure S5: GC–MS chromatograms of (A) acidic fraction and (B) neutral fraction of sample IF 1906. The peaks are labeled according to Table 2. Figure S6: SEM–EDS spot analysis of sample IF 1906 showing the presence of lead. Figure S7: GC–MS chromatograms of (A) acidic fraction and (B) neutral fraction of sample IF 1916. The peaks are labeled according to Table 2. (*): contaminants. Figure S8: SEM–EDS spot analysis of sample IF 1916 showing the presence of calcium. Figure S9: SEM–EDS spot analysis of sample IF 1916 showing the presence of zirconium. Figure S10: SEM–EDS spot analysis of sample IF 1916 showing the presence of gold. Figure S11: GC–MS chromatograms of (A) acidic fraction and (B) neutral fraction of sample IF 2405. The peaks are labeled according to Table 2. Figure S12: GC–MS chromatograms of (A) acidic fraction and (B) neutral fraction of sample IF 341. The peaks are labeled according to Table 2. Figure S13: GC–MS chromatograms of (A) acidic fraction and (B) neutral fraction of sample IF1857. The peaks are labeled according to Table 2. Figure S14: GC–MS chromatograms of (A) acidic fraction and (B) neutral fraction of sample IF993. The peaks are labeled according to Table 2. Figure S15: GC–MS chromatograms of (A) acidic fraction and (B) neutral fraction of sample IF698. The peaks are labeled according to Table 2. Figure S16: GC–MS chromatograms of (A) acidic fraction and (B) neutral fraction of sample IF323. The peaks are labeled according to Table 2. Figure S17: GC–MS chromatograms of (A) acidic fraction and (B) neutral fraction of sample IF1317. The peaks are labeled according to Table 2. Figure S18: GC–MS chromatograms of (A) acidic fraction and (B) neutral fraction of sample IF2407. The peaks are labeled according to Table 2. Figure S19: Pyrograms of samples IF 698 (red) and IF 1317 (blue). TMS: trimethylsilyl derivatives. *: HMDS pyrolysis product.
