# Supplementary material for: Historical Drugs in Transylvania: Disclosing the Composition of Ointments from the “History of Pharmacy Collection” in Cluj-Napoca Through a Multi-Analytical Approach
Source: Molecules. 2024 Nov 14;29(22):5356. doi: 10.3390/molecules29225356 (PMC11596852; doi:10.3390/molecules29225356)
Supplement: Supplementary file 1 [file molecules-29-05356-s001.zip › molecules-3261540-supplementary.pdf]

Supplementary material

Historical Drugs in Transylvania: Disclosing the Composition of Ointments from the "History of Pharmacy Collection" in Cluj-Napoca Through a Multi-Analytical Approach

Federica Nardella [1,*], Jacopo La Nasa [1], Ilaria Degano [1], Francesca Modugno [1], Ana-Maria Gruia [2], Ioana Cova [2], Andrea Beatrix Magó [2], Márta Guttmann [3] and Erika Ribechini [1]

1 Department of Chemistry and Industrial Chemistry, University of Pisa, I-56123 Pisa, Italy; jacopo.lanasa@unipi.it (J.L.N.); ilaria.degano@unipi.it (I.D.); francesca.modugno@unipi.it (F.M.); erika.ribechini@unipi.it (E.R.)
2 National Museum of Transylvanian History, 400020 Cluj-Napoca, Romania; ana.gruia@gmail.com (A.-M.G.); ioanatescancova@yahoo.com (I.C.); beatrix.b3@gmail.com (A.B.M.)
3 Department of History, Heritage and Protestant Theology, Faculty of Social and Human Sciences, "Lucian Blaga" University of Sibiu, 550024 Sibiu, Romania; marta.guttmann@ulbsibiu.ro
* Correspondence: federica.nardella@dcci.unipi.it

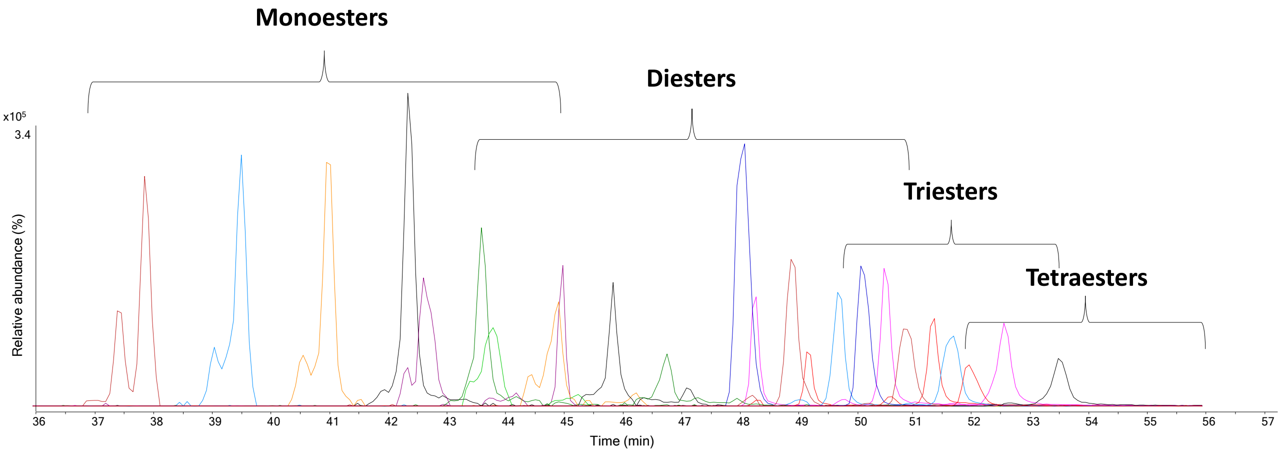

Figure S1: EICs profiles obtained from HPLC-MS/MS analysis of the extract of sample IF 1908. Profiles are obtained by overlapping EICs corresponding to the m/z of [M+Na]+ reported in literature [17] for the different species .

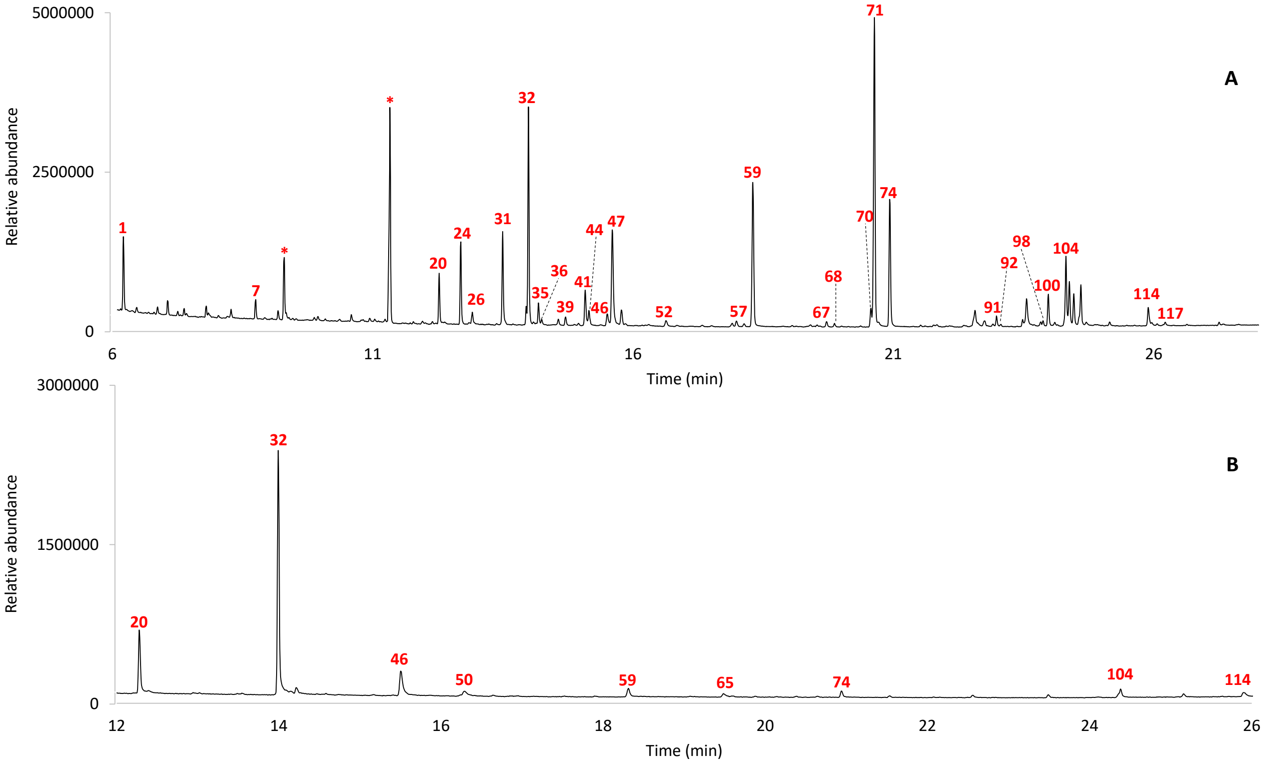

Figure S2: GC-MS chromatograms of (A) acidic fraction and (B) neutral fraction of sample IF 1918. The peaks are labeled according to Table 2. (*): contaminants.

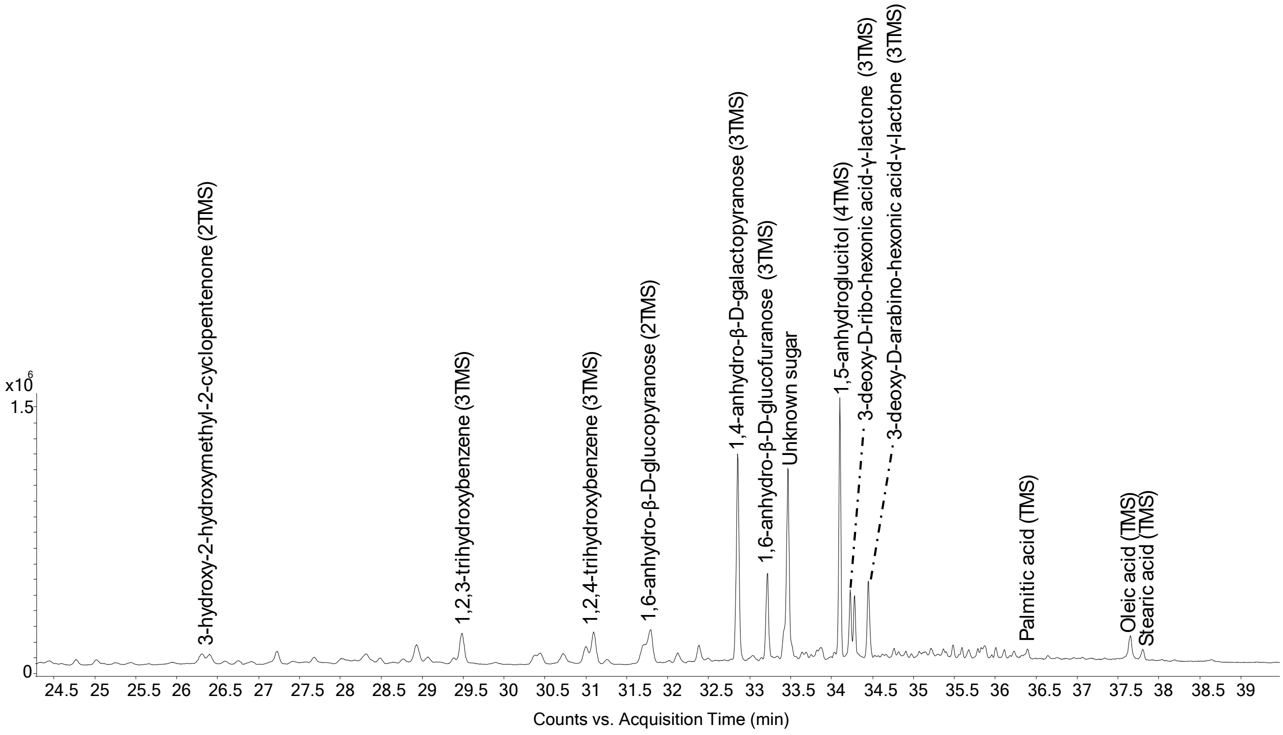

Figure S3: Pyrogram of sample IF 1918. TMS: trimethylsilyl derivatives.

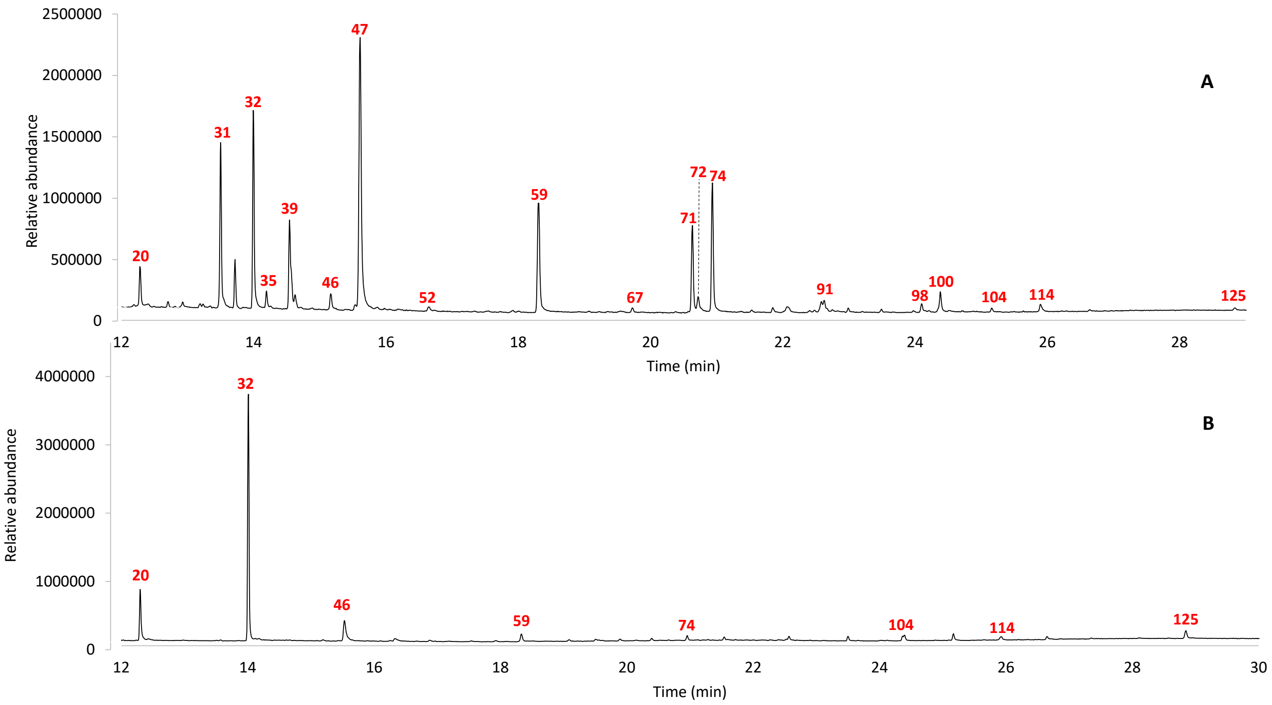

Figure S4: GC-MS chromatograms of (A) acidic fraction and (B) neutral fraction of sample IF 1914. The peaks are labeled according to Table 2.

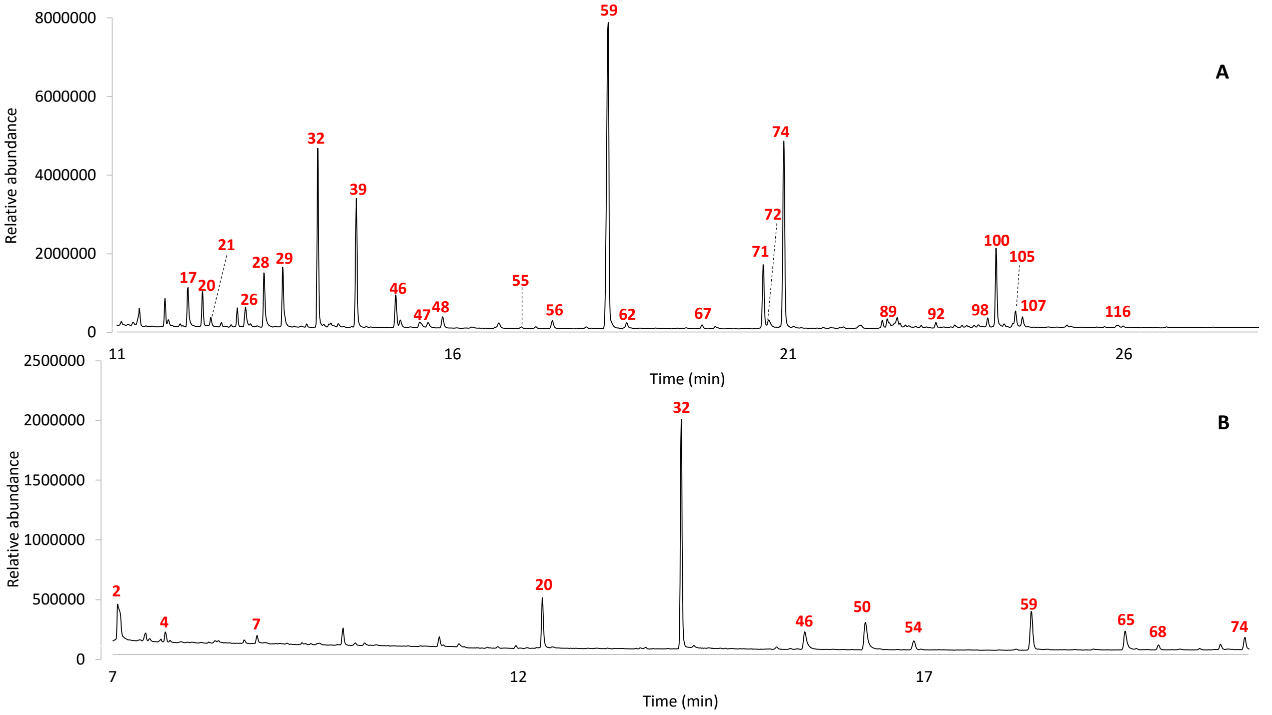

Figure S5: GC-MS chromatograms of (A) acidic fraction and (B) neutral fraction of sample IF 1906. The peaks are labeled according to Table 2.

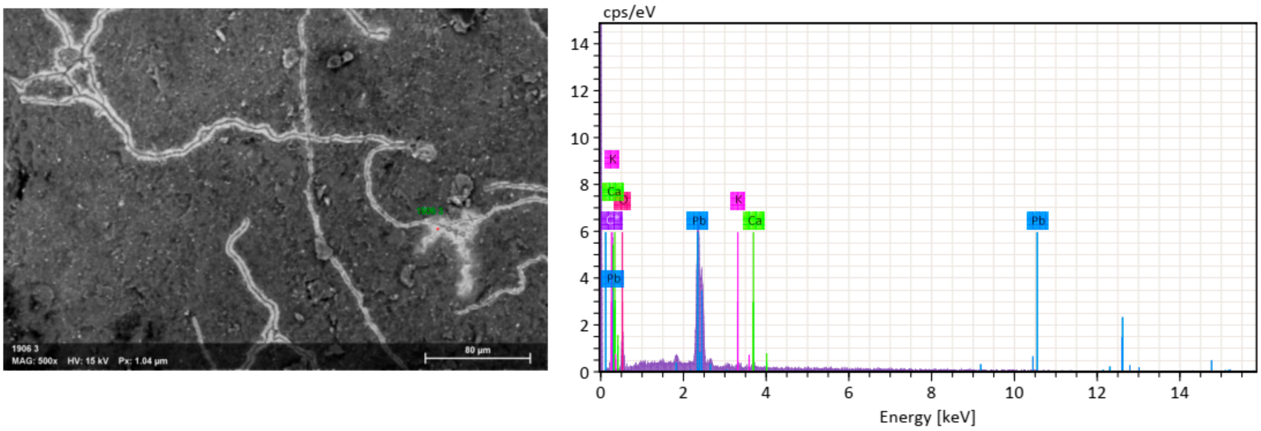

1906 3

Element	At. No.	Netto	Mass [%]	Mass Norm. [%]	Atom [%]	abs. error [%] (1 sigma)
Carbon	6	3694	0.00	0.00	0.00	0.00
Oxygen	8	1405	15.68	19.24	72.90	3.27
Potassium	19	105	0.42	0.52	0.80	0.07
Calcium	20	394	1.88	2.31	3.50	0.15
Lead	82	14005	63.51	77.93	22.80	2.36
		**Sum**	**81.50**	**100.00**	**100.00**	

Figure S6: SEM-EDS spot analysis of sample IF 1906 showing the of presence of lead.

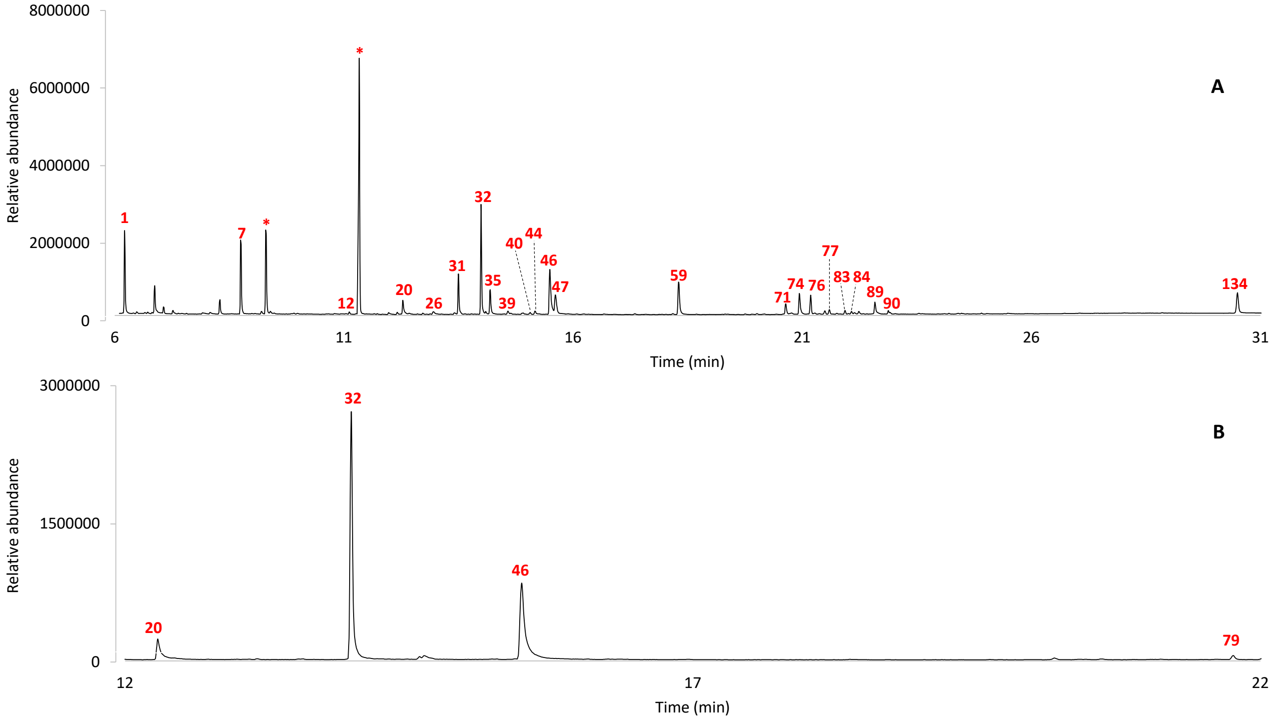

Figure S7: GC-MS chromatograms of (A) acidic fraction and (B) neutral fraction of sample IF 1916. The peaks are labeled according to Table 2. (*): contaminants.

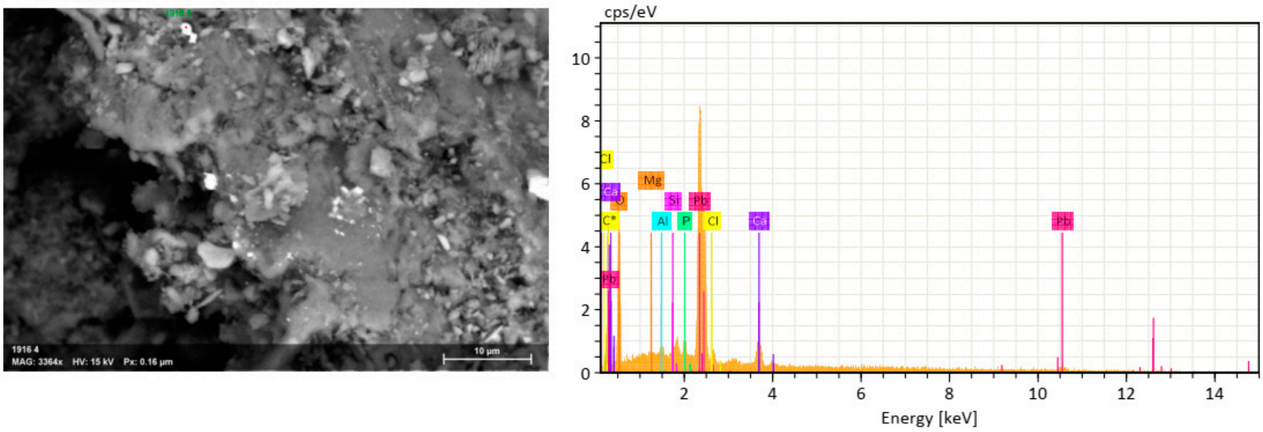

1916 5

Element	At. No.	Netto	Mass [%]	Mass Norm. [%]	Atom [%]	abs. error [%] (1 sigma)
Carbon	6	2931	0.00	0.00	0.00	0.00
Oxygen	8	4095	25.21	29.73	77.48	4.12
Magnesium	12	103	0.16	0.19	0.33	0.05
Aluminium	13	338	0.48	0.57	0.88	0.07
Silicon	14	313	0.40	0.48	0.71	0.06
Phosphorus	15	687	0.89	1.05	1.42	0.08
Chlorine	17	167	0.34	0.40	0.47	0.06
Calcium	20	1649	5.17	6.10	6.34	0.24
Lead	82	18844	52.14	61.49	12.37	1.92
		**Sum**	**84.79**	**100.00**	**100.00**	

Figure S8: SEM-EDS spot analysis of sample IF 1916 showing the of presence of calcium.

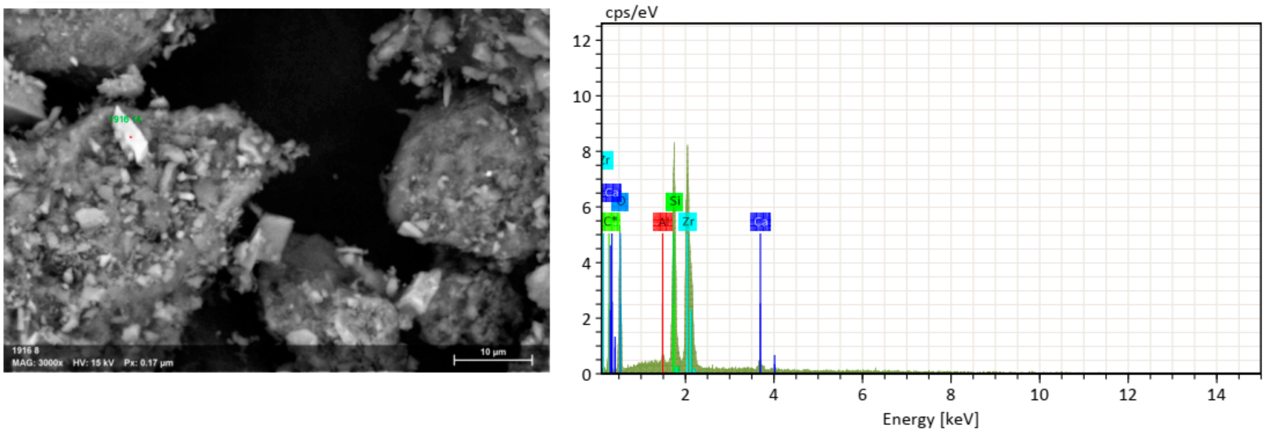

1916 14

Element	At. No.	Netto	Mass [%]	Mass Norm. [%]	Atom [%]	abs. error [%] (1 sigma)
Carbon	6	1760	0.00	0.00	0.00	0.00
Oxygen	8	5438	33.11	35.03	66.24	5.13
Aluminium	13	403	0.48	0.51	0.57	0.06
Silicon	14	12334	13.94	14.74	15.88	0.63
Calcium	20	777	1.83	1.94	1.46	0.12
Zirconium	40	20347	45.18	47.79	15.85	1.76
		**Sum**	**94.54**	**100.00**	**100.00**	

Figure S9: SEM-EDS spot analysis of sample IF 1916 showing the of presence of zirconium.

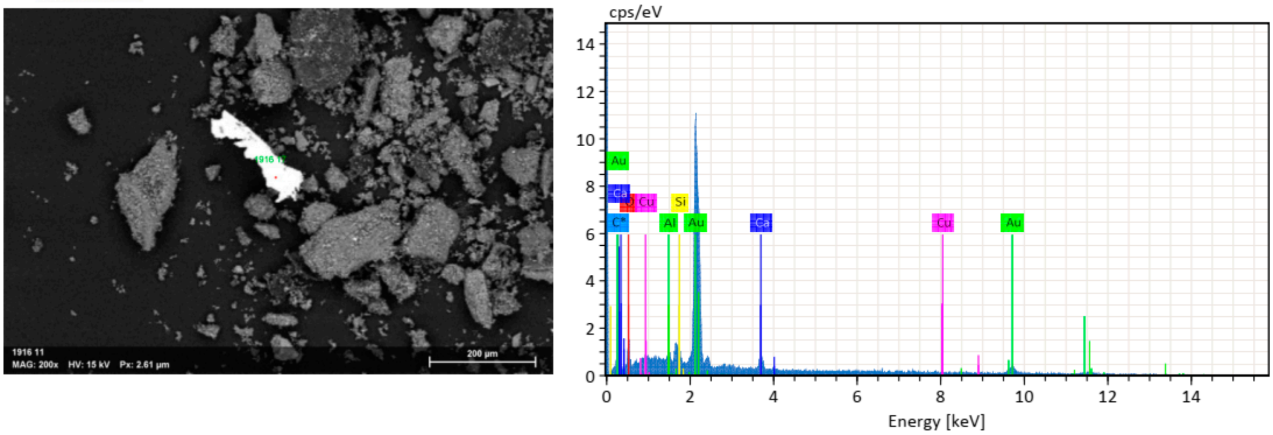

1916 17

Element	At. No.	Netto	Mass [%]	Mass Norm. [%]	Atom [%]	abs. error [%] (1 sigma)
Carbon	6	3842	0.00	0.00	0.00	0.00
Oxygen	8	1171	3.83	5.83	36.14	0.86
Aluminium	13	431	0.47	0.72	2.63	0.06
Silicon	14	597	0.60	0.91	3.21	0.07
Calcium	20	1366	3.69	5.62	13.92	0.18
Copper	29	17	0.19	0.28	0.44	0.07
Gold	79	28559	56.94	86.64	43.65	2.20
		Sum	65.71	100.00	100.00	

Figure S10: SEM-EDS spot analysis of sample IF 1916 showing the of presence of gold.

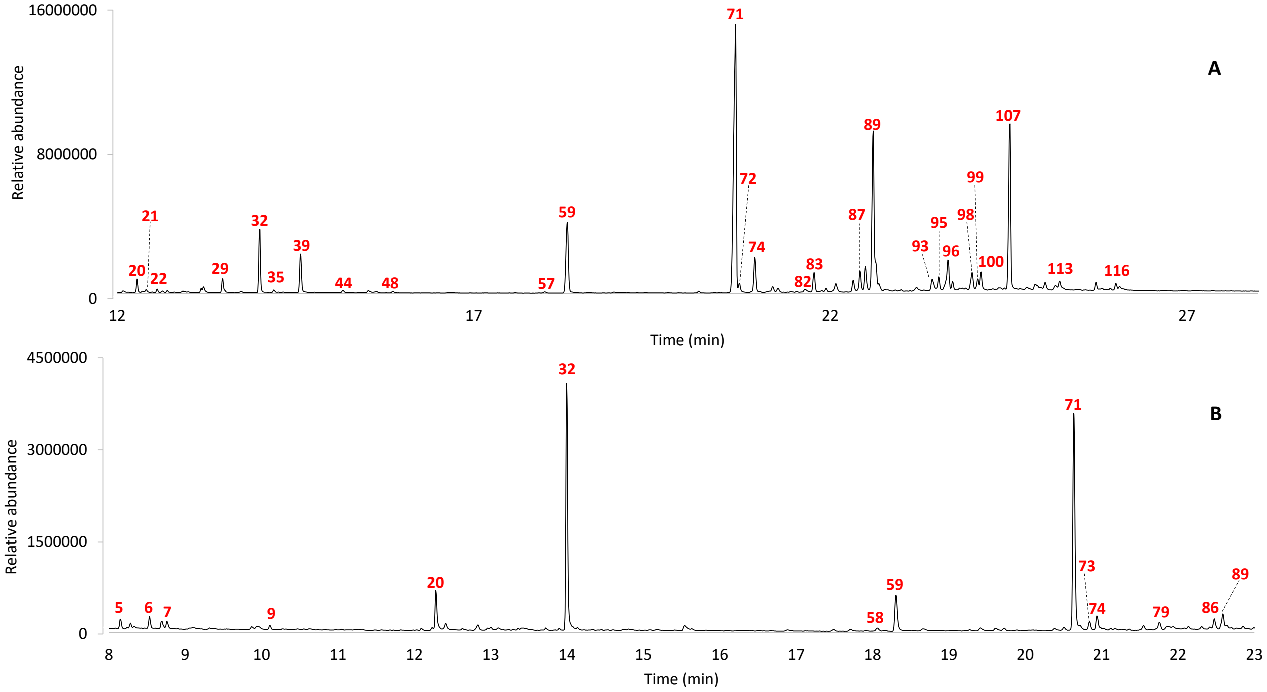

Figure S11: GC-MS chromatograms of (A) acidic fraction and (B) neutral fraction of sample IF 2405. The peaks are labeled according to Table 2.

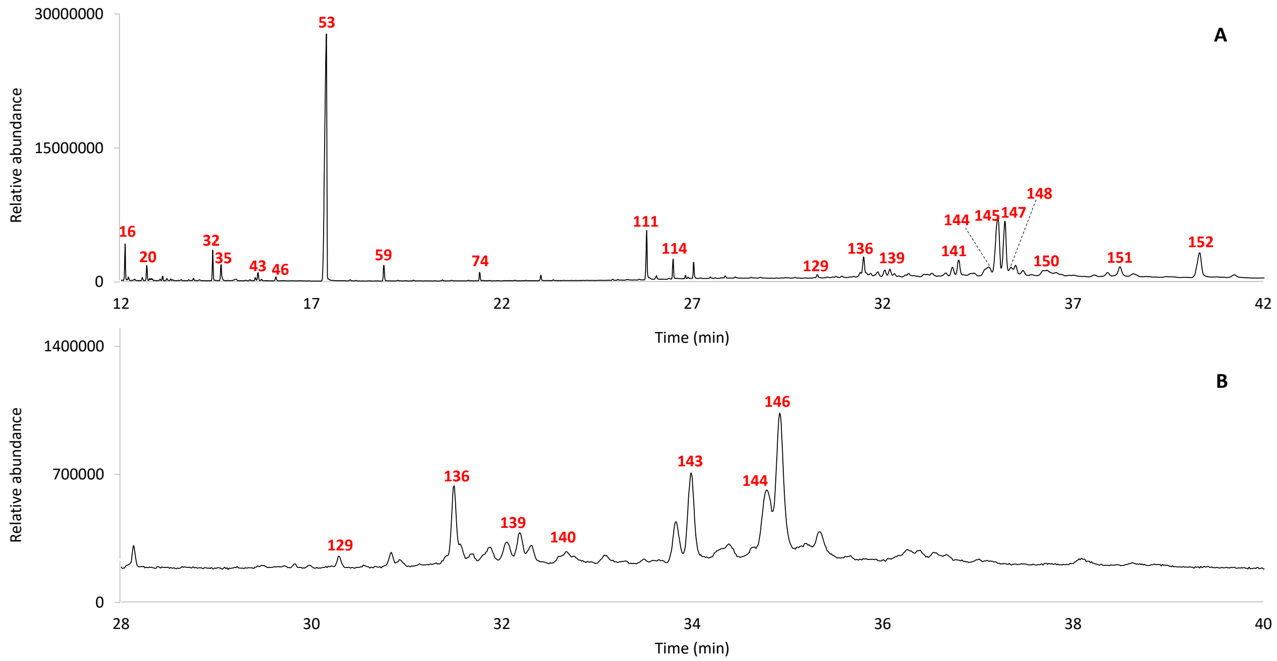

Figure S12: GC-MS chromatograms of (A) acidic fraction and (B) neutral fraction of sample IF 341. The peaks are labeled according to Table 2.

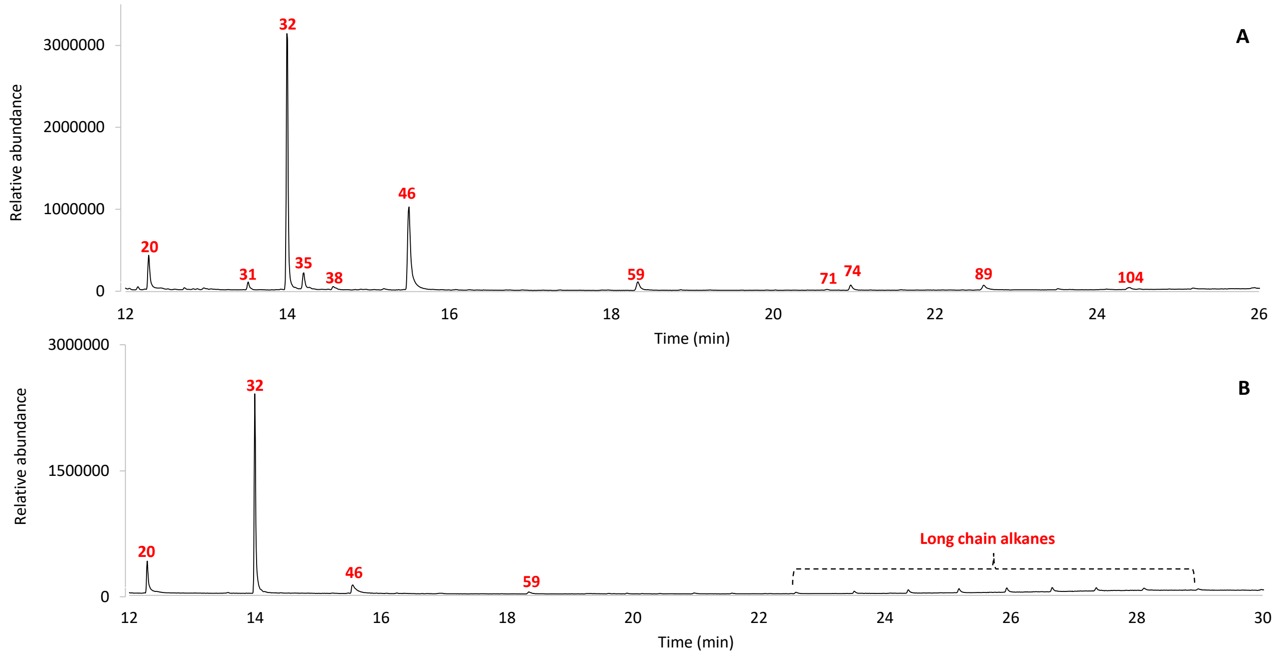

Figure S13: GC-MS chromatograms of (A) acidic fraction and (B) neutral fraction of sample IF1857. The peaks are labeled according to Table 2.

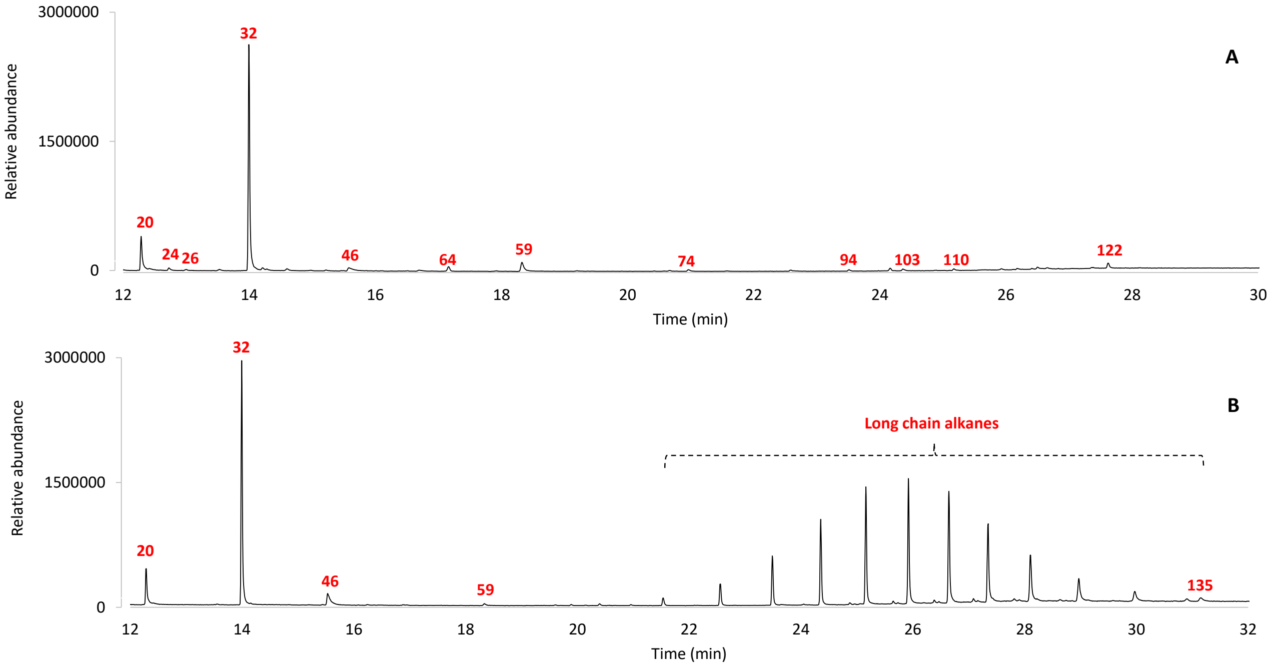

Figure S14: GC-MS chromatograms of (A) acidic fraction and (B) neutral fraction of sample IF993. The peaks are labeled according to Table 2.

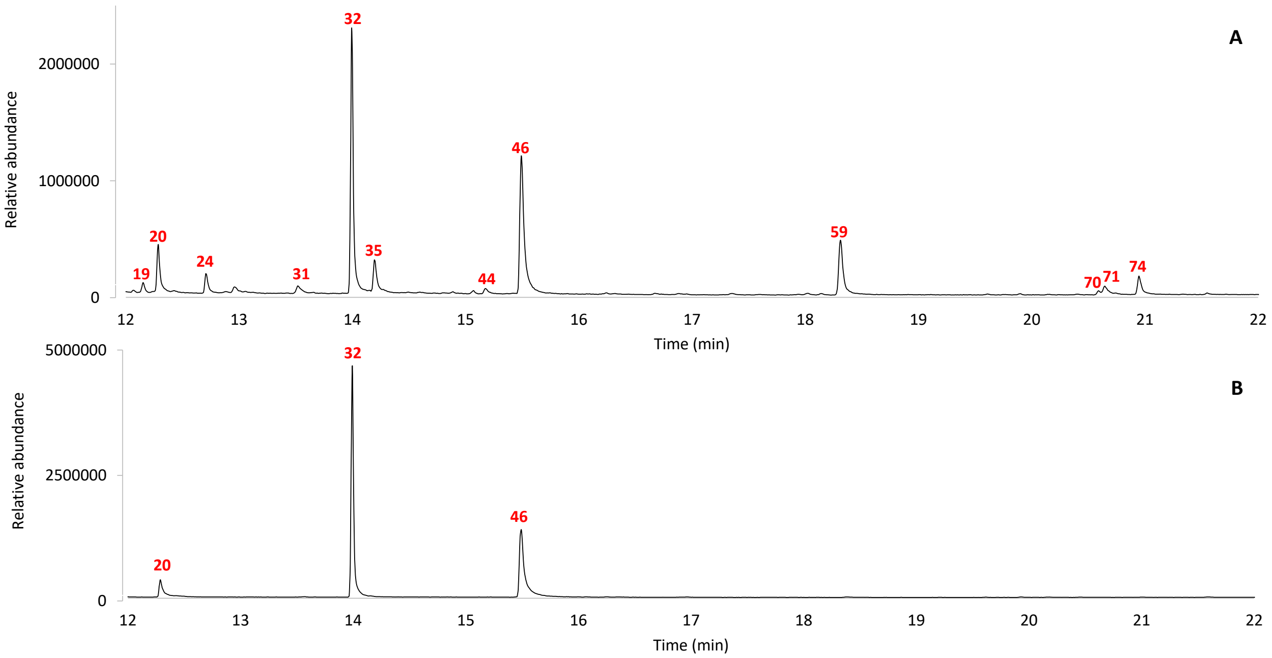

Figure S15: GC-MS chromatograms of (A) acidic fraction and (B) neutral fraction of sample IF698. The peaks are labeled according to Table 2.

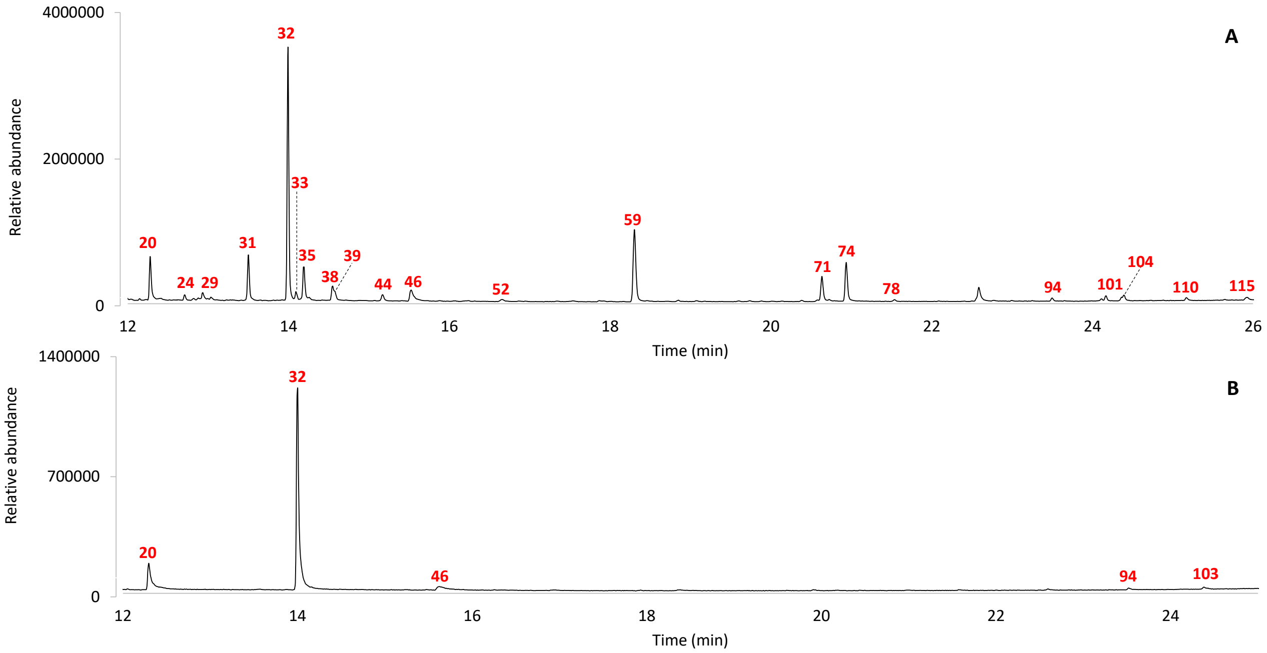

Figure S16: GC-MS chromatograms of (A) acidic fraction and (B) neutral fraction of sample IF323. The peaks are labeled according to Table 2.

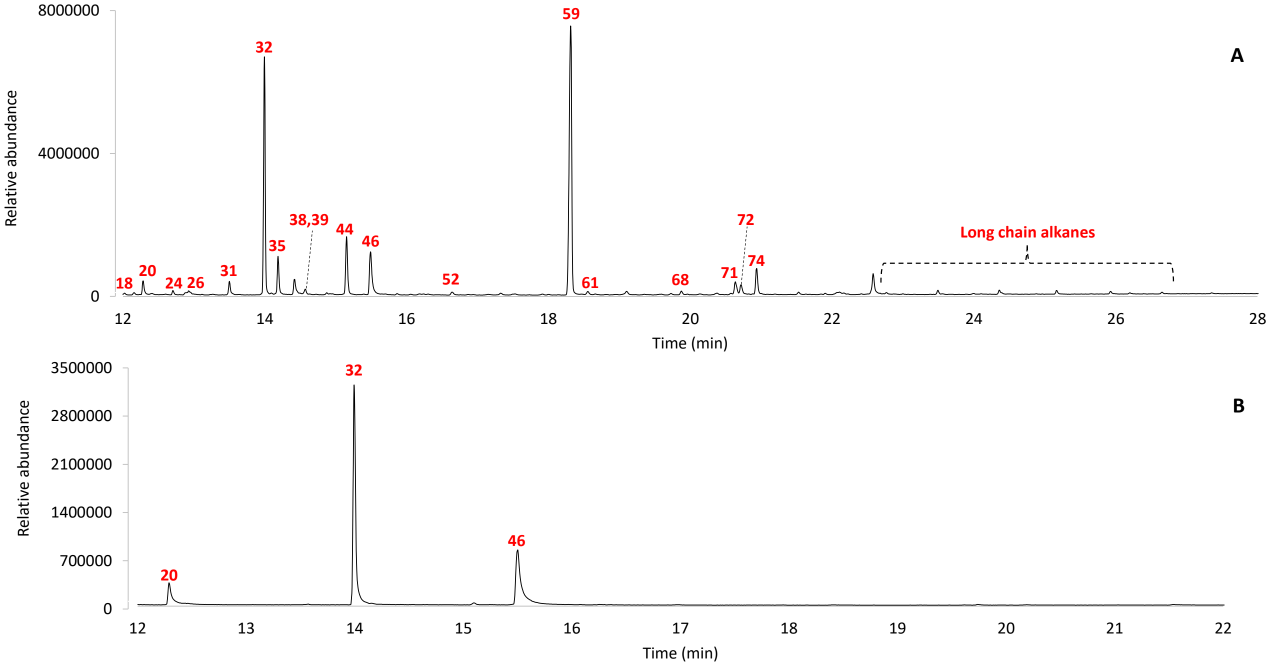

Figure S17: GC-MS chromatograms of (A) acidic fraction and (B) neutral fraction of sample IF1317. The peaks are labeled according to Table 2.

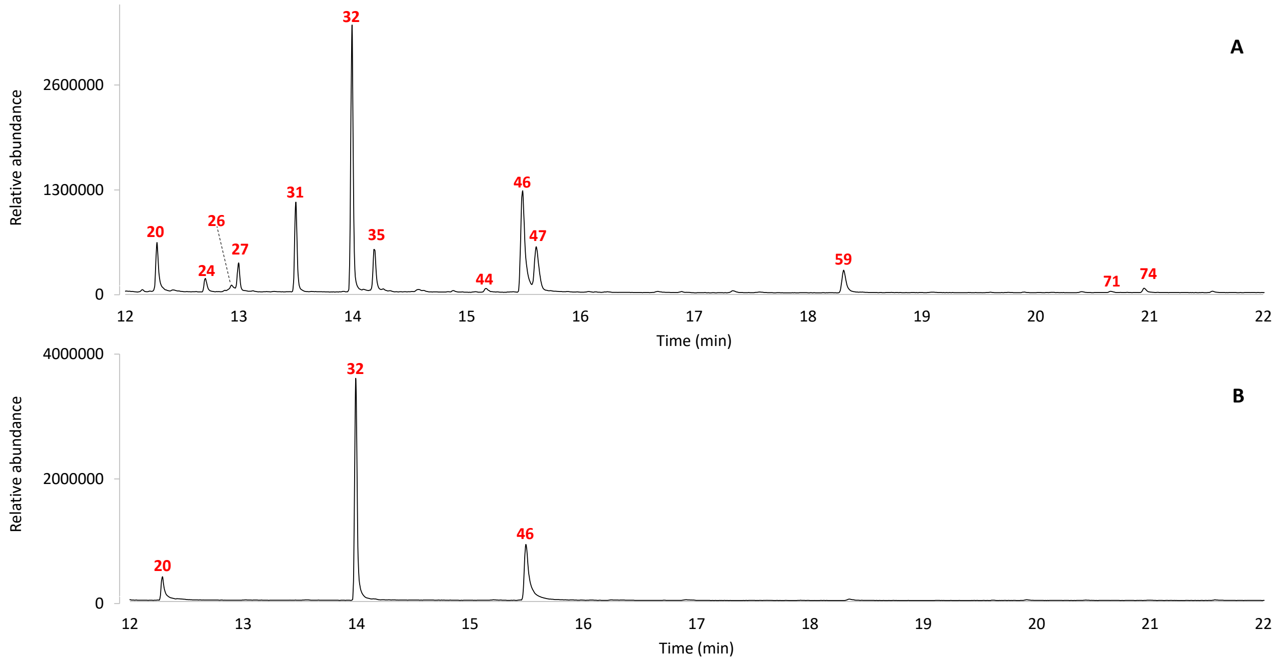

Figure S18: GC-MS chromatograms of (A) acidic fraction and (B) neutral fraction of sample IF2407. The peaks are labeled according to Table 2.

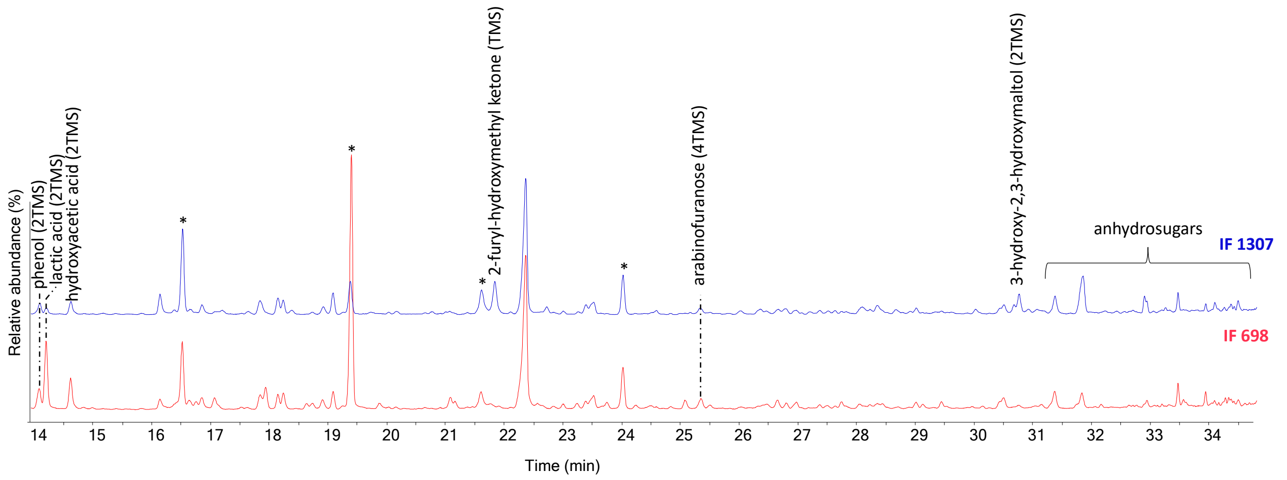

Figure S19: Pyrograms of samples IF 698 (red) and IF 1317 (blue). TMS: trimethylsilyl derivatives. *: HMDS pyrolysis product.
